# Benchmarking Long-Read Assemblers for Genomic Analyses of Bacterial Pathogens Using Oxford Nanopore Sequencing

**DOI:** 10.3390/ijms21239161

**Published:** 2020-12-01

**Authors:** Zhao Chen, David L. Erickson, Jianghong Meng

**Affiliations:** Joint Institute for Food Safety and Applied Nutrition, Center for Food Safety and Security Systems, Department of Nutrition and Food Science, University of Maryland, College Park, MD 20742, USA; zhchen29@umd.edu (Z.C.); derickso@umd.edu (D.L.E.)

**Keywords:** whole-genome sequencing, long-read sequencing, Oxford Nanopore sequencing, genome assembly, long-read assembler, genomic analysis, bacterial pathogen, benchmarking

## Abstract

Oxford Nanopore sequencing can be used to achieve complete bacterial genomes. However, the error rates of Oxford Nanopore long reads are greater compared to Illumina short reads. Long-read assemblers using a variety of assembly algorithms have been developed to overcome this deficiency, which have not been benchmarked for genomic analyses of bacterial pathogens using Oxford Nanopore long reads. In this study, long-read assemblers, namely Canu, Flye, Miniasm/Racon, Raven, Redbean, and Shasta, were thus benchmarked using Oxford Nanopore long reads of bacterial pathogens. Ten species were tested for mediocre- and low-quality simulated reads, and 10 species were tested for real reads. Raven was the most robust assembler, obtaining complete and accurate genomes. All Miniasm/Racon and Raven assemblies of mediocre-quality reads provided accurate antimicrobial resistance (AMR) profiles, while the Raven assembly of *Klebsiella variicola* with low-quality reads was the only assembly with an accurate AMR profile among all assemblers and species. All assemblers functioned well for predicting virulence genes using mediocre-quality and real reads, whereas only the Raven assemblies of low-quality reads had accurate numbers of virulence genes. Regarding multilocus sequence typing (MLST), Miniasm/Racon was the most effective assembler for mediocre-quality reads, while only the Raven assemblies of *Escherichia coli* O157:H7 and *K. variicola* with low-quality reads showed positive MLST results. Miniasm/Racon and Raven were the best performers for MLST using real reads. The Miniasm/Racon and Raven assemblies showed accurate phylogenetic inference. For the pan-genome analyses, Raven was the strongest assembler for simulated reads, whereas Miniasm/Racon and Raven performed the best for real reads. Overall, the most robust and accurate assembler was Raven, closely followed by Miniasm/Racon.

## 1. Introduction

The rapid development of whole-genome sequencing (WGS) technologies over the last decade has revolutionized the ability to monitor and trace outbreaks of infectious diseases [1]. Illumina short-read sequencing has been widely used for sequencing bacterial pathogens, which can produce millions of short reads (<300 bp) in length and low error rates of 1–2% [2]. A high-quality bacterial genome assembly should be both accurate and complete based on the concept of “one chromosome, one contig” proposed by Koren and Phillippy [3]. However, Illumina sequencing is generally insufficient for assembling complete genomic structures to investigate how genes are organized within genomes and whether these genes are located on the chromosome or plasmids of bacteria [4]. This limitation is mainly attributed to the fact that short reads are not able to span the repetitive structures that extend beyond the maximum read length generated, thus producing unresolvable loops during genome assembly and resulting in an assembly consisting of many unordered contigs. Moreover, Illumina sequencing has difficulties in resolving GC-rich regions, making it problematic to reconstruct these genetic regions [5].

One primary solution to overcome this deficiency is to use long reads such as those generated by Oxford Nanopore sequencing [6]. The primary limitation of Oxford Nanopore sequencing is that the error (single-nucleotide polymorphisms [SNPs] and indels) rates are greater compared to Illumina sequencing, although they have decreased in recent years due to the continued improvements in nanopore chemistry and basecalling [7]. Given the value of Oxford Nanopore long reads, significant effort has been made with computational approaches to incorporate long reads into assembly algorithms while mitigating the high error rates [8].

Multiple long-read assemblers using a variety of assembly algorithms such as Canu, Flye, Miniasm, Raven, Redbean, and Shasta are currently available. Canu involves a modified string graph algorithm, often referred to as the overlap–layout–consensus (OLC) approach [9]. It first corrects long reads, then removes adapters and breaks chimeras, and finally assembles the reads into contigs.

Flye works by combining long reads into error-prone ‘disjointigs’, using the repetitive sequences to generate a repeat graph, and then resolving the repeats to produce the final assembly [10,11].

Miniasm is an ultrafast OLC-based assembler for long reads [12]. It takes all-vs-all read self-mappings as input by minimap2 and simply concatenates the read sequences to generate the final unitig, resulting in a per-base error rate similar to that of the raw reads. However, in contrast to other assemblers, Miniasm does not include a consensus step. A polishing tool such as Racon [13] should, therefore, be used to achieve high accuracy through the agreement between the reads and the assembly following the initial Miniasm assembly.

Raven is another assembler that uses the OLC approach [14]. It first finds overlaps between long reads by chaining the minimizer hits with minimap2, then creates an assembly graph and simplifies it using a submodule from Rala, and finally polishes the obtained contigs with a partial order alignment submodule from Racon.

Redbean, also known as Wtdbg2, employs a long-read assembly approach called a fuzzy de Bruijn graph, which distinguishes it from the majority of other long-read assemblers [15]. Redbean decomposes long reads into 1024 bp segments, merges similar segments into a vertex, and connects vertices based on the segment adjacency on the reads to produce a fuzzy de Bruijn graph. It is analogous to the de Bruijn graph used for the short-read assembly but modified to permit mismatches/gaps present in the noisy long reads and keeps the read paths when collapsing k-mers.

Shasta with a high computational efficiency uses a run-length representation of the long-read sequences [16], which makes the assembly more resilient to errors in the homopolymer repeat counts commonly found in Oxford Nanopore long reads. The assembly using Shasta is accomplished not directly on the long reads but rather on a fixed subset of short k-mers (k ≈ 10) used to develop an assembly graph using a consensus sequence.

As the advantages of Oxford Nanopore sequencing over PacBio sequencing are the longer read length, the higher throughput, and the lower costs [6], Oxford Nanopore sequencing can play an increasingly critical role in bacterial genomics. The importance of robust and reliable long-read assemblers will continue to grow. The selection of an effective long-read assembly approach has significant implications for the identification, genotypic prediction, genome-wide annotation, and phylogenetic inference of bacterial pathogens. Wick and Holt [17] assessed the performance of six long-read assemblers (Canu, Flye, Miniasm/Minipolish, Raven, Redbean, and Shasta) on long reads of bacteria in terms of their structural completeness and accuracy, sequence identity, contig circularization, and computational resources used. They found each of the assemblers tested had pros and cons, and while there was no ideal assembler for assembling prokaryote genomes using long reads, Flye, Miniasm/Minipolish, and Raven were observed to the overall best performers. However, to our knowledge, no benchmarking of these long-read assemblers for downstream genomic analyses of bacterial pathogens using Oxford Nanopore sequencing has been performed.

In the present study, we assessed the aforementioned long-read assemblers in the context of Oxford Nanopore sequencing of major bacterial pathogens. The selected species covered a wide range of genome sizes and GC contents. We evaluated and compared the capabilities of these assemblers in generating complete and accurate assemblies in terms of genome completeness and accuracy, antimicrobial resistance (AMR), virulence potential, phylogeny, and pan genome. Specifically, we utilized both simulated (mediocre- and low-quality) and real reads for selected species of bacterial pathogens. Simulated reads allow for confident ground truth, as the underlying reference genome is known with certainty. Alternatively, the use of real reads can better inform us of how real reads interact with the assembly algorithms to produce accurate genomic assemblies. By taking into consideration both types of data on species with different genomic characteristics, we can better evaluate the underlying algorithms of each assembler, and how they may affect the completeness and accuracy of genome assemblies and downstream genomic analyses.

## 2. Results and Discussion

### 2.1. Genome Completeness and Accuracy

For simulated reads which contained artificial error profiles, genome completeness and accuracy indicate the robustness of an assembler to tolerate a wide range of read parameters such as chimeras, low-quality regions, and systematic basecalling errors [17]. The Canu, Miniasm/Racon, and Raven assemblies of mediocre-quality reads had numbers of contigs not significantly different (*p* > 0.05) from the reference genomes (Table 1), while Flye, Redbean, and Shasta produced significantly higher (*p* < 0.05) numbers of contigs than the reference genomes. The Redbean assemblies generated genome sizes significantly larger than the reference genomes (*p* < 0.05), whereas other assemblers produced assemblies with significantly lower (*p* < 0.05) genome sizes than the reference genomes. The GC contents of all assemblies were not significantly different (*p* > 0.05) from those of the reference genomes. The complete benchmarking universal single-copy orthologs (BUSCOs) of all assemblies were significantly lower (*p* < 0.05) than those of the reference genomes. Significantly higher (*p* < 0.05) complete BUSCOs were observed in the Miniasm/Racon and Raven assemblies compared to other assemblers (Supplementary Table S1), while the complete BUSCOs of the Raven assemblies were significantly higher (*p* < 0.05) than those of the Miniasm/Racon assemblies. Significantly lower (*p* < 0.05) numbers of SNPs and indels were detected in the Canu, Miniasm/Racon, and Raven assemblies than other assemblies (Supplementary Table S2). The numbers of SNPs and indels of the Canu and Raven assemblies were not significantly different (*p* > 0.05) but were significantly lower (*p* < 0.05) than the Miniasm/Racon assemblies.

We also tested simulated Oxford Nanopore long reads of low quality to examine whether each long-read assembler could tolerate a higher degree of errors. When assembling low-quality reads, Canu and Shasta failed to generate assemblies for all strains (Table 2), while Flye and Redbean did not produce assemblies for three and one out of 10 strains, respectively. Similarly, Wick and Holt (2020) also reported the incomplete assembly processes of Canu, Flye, Redbean, and Shasta when simulated reads of prokaryote genomes were tested. In contrast, Miniasm/Racon and Raven generated assemblies for all strains. Compared to mediocre-quality reads, Flye, Miniasm/Racon, Redbean, and Raven produced assemblies with more fragmented contigs using low-quality reads. The numbers of contigs of the Raven and Redbean assemblies were not significantly different (*p* > 0.05) from those of the reference genomes, whereas Flye and Miniasm/Racon generated significantly more (*p* < 0.05) contigs than the reference genomes. The genome sizes and GC contents of the Raven assemblies were still not significantly different (*p* > 0.05) from those of the reference genomes, although low-quality reads were used. Flye, Miniasm/Racon, and Redbean all produced significantly different (*p* < 0.05) GC contents from the reference genomes. The complete BUSCOs of the Raven assemblies were still significantly higher (*p* < 0.05) than those of the Flye, Miniasm/Racon, and Redbean assemblies (Supplementary Table S3). As Flye and Miniasm/Racon produced assemblies with inaccurate genome sizes using low-quality reads, the numbers of SNPs and indels of the assemblies of low-quality reads were not compared with those of mediocre-quality reads. Significant increases (*p* < 0.05) in the numbers of SNPs and indels were observed in the Raven assemblies of low-quality reads compared to mediocre-quality reads (Supplementary Table S4).

Compared to when mediocre-quality reads were tested, the numbers of contigs of the Flye, Miniasm/Racon, Raven, and Redbean assemblies of low-quality reads became significantly higher (*p* < 0.05) when using low-quality reads. No significant differences (*p* > 0.05) were observed between the genome sizes of the Raven assemblies of mediocre- and low-quality reads, while the genome sizes of the Flye, Miniasm/Racon, and Redbean assemblies of low-quality reads were significantly lower (*p* < 0.05) than those of mediocre-quality reads. There were no significant differences (*p* > 0.05) in GC content between the Flye, Miniasm/Racon, Raven, and Redbean assemblies of mediocre- and low-quality reads. The complete BUSCOs of the Flye, Miniasm/Racon, Raven, and Redbean assemblies of low-quality reads were significantly lower than those of mediocre-quality reads (*p* < 0.05), while significantly higher (*p* < 0.05) numbers of SNPs and indels were observed when low-quality reads were tested.

Despite the advantages of simulated reads in allowing for a controlled evaluation of the effect of long-read quality on genome assembly, they cannot completely capture the challenges of using real Oxford Nanopore reads when assessing long-read assemblers. The genome completeness and accuracy of an assembler indicate its reliability to achieve a complete and accurate assembly given a set of real reads, which incorporate naturally occurring features of Oxford Nanopore long reads (e.g., error profiles, read lengths, quality scores) [17]. All assemblers completed assembly processes for each strain to generate assemblies when real reads were used (Table 3). Canu, Flye, and Raven were superior to other assemblers, and produced the most contiguous assemblies, while other assemblers produced inaccurate numbers of contigs for some strains. For example, Miniasm/Racon produced 227 and 43 contigs for *Clostridium botulinum* CFSAN034200 and *C. jejuni* NCTC 11168, respectively. Shasta generated 850 contigs for *C. sakazakii* CFSAN068773. Redbean performed poorly in achieving contiguous assemblies, often producing inaccurate numbers of contigs. The genome sizes and GC contents of the Miniasm/Racon, Raven, Redbean, and Shasta assemblies were not significantly different (*p* > 0.05) from those of the reference genomes, whereas Canu and Flye failed to produce assemblies which had genome sizes and GC contents not significantly different from the reference genomes (*p* > 0.05). The complete BUSCOs of all assemblies were significantly lower (*p* < 0.05) than those of the reference genomes (Supplementary Table S5). The Miniasm/Racon and Raven assemblies had the highest (*p* < 0.05) complete BUSCOs compared to other assemblers, while no significant differences (*p* > 0.05) were observed between the Miniasm/Racon and Raven assemblies. The numbers of SNPs of the Canu assemblies were significantly lower (*p* < 0.05) than those of the assemblies produced by other assemblers (Supplementary Table S6), while no significant differences (*p* > 0.05) were found among other assemblers, with only one exception that the Raven assemblies had the numbers of SNPs significantly lower (*p* < 0.05) than the Miniasm/Racon assemblies. The Raven assemblies had the lowest (*p* < 0.05) numbers of indels among all assemblers.

As predicted with the aid of the PlasmidFinder database, the assemblies of simulated reads showed inconsistent plasmid profiles (types and numbers of plasmids) with the reference genomes in some cases (Supplementary Tables S7 and S8). All assemblies of *Bacillus anthracis* Ames Ancestor showed consistent plasmid profile with the reference genome. Only the Flye assembly of *S.* Typhimurium LT2 contained both IncFIB (S) and IncFII (S), which was consistent with the reference genome. The plasmid profiles of the Redbean and Shasta assemblies of *Cronobacter sakazakii* ATCC 29544 were consistent with that of the reference genome. None of the assemblies of *Escherichia coli* O157:H7 Sakai carried IncFIB (AP001918) that was present in the reference genome. Among the assemblies of low-quality reads, the Raven assembly of *Staphylococcus aureus* TW20 was the only one that carried a plasmid, whereas plasmids were not present in other assemblies.

Similarly, plasmid profiles inconsistent with the reference genomes were also observed in the assemblies of real reads (Supplementary Table S9). Compared to the reference genomes, the Shasta assembly of *S.* Bareilly CFSAN000189 did not harbor IncFII (S). The Flye assembly of *S. aureus* CFSAN007894 had a consistent plasmid profile with the reference genome, while inconsistent plasmid profiles were identified in other assemblies. Interestingly, similar to *Escherichia coli* O157:H7 Sakai with simulated reads, none of the assemblies of *E. coli* O157:H7 CFSAN076619 possessed IncFIB (AP001918) that was contained in the reference genome, which could be attributed to the loss of this plasmid during Oxford Nanopore library preparation. This finding also pointed to the suboptimal library preparation bias as a second possible explanation [18]. Consequently, there is a current need for reliable protocols to extract high-molecular-weight genomic DNA from bacteria that are compatible with Oxford Nanopore sequencing to overcome this deficiency. The degree to which Oxford Nanopore sequencing is compatible with diverse DNA extraction chemistries remains to be validated.

According to the assemblies using both simulated and real reads, overall, Raven emerged as the best assembler that can be tolerant of both low-identity and realistic reads. Noticeably, Raven was the most robust assembler that was not significantly affected by random reads, junk reads, or chimeric reads in low-quality reads. It should be noted that one expected benefit of Oxford Nanopore sequencing is that it can identify the exact locations of mobile genetic elements (MGEs) such as plasmids. Unfortunately, since Raven does not indicate the circularity of contigs that harbor plasmids, it is not able to determine whether plasmids are integrated into the chromosome or exist extra-chromosomally in a non-integrated state. This drawback of Raven may potentially cause an imperfect representation of the genetic architectures of MGEs.

### 2.2. Antimicrobial Resistance Genes (ARGs)

Oxford Nanopore sequencing has great potential for accelerating AMR genotyping, as sequence data are acquired in real time within minutes of sequencing [19,20]. Meanwhile, Oxford Nanopore sequencing enables the identification of MGEs on which ARGs are located and also characterizes the combination of different ARGs co-located on the same MGEs. However, it is worth noting that Oxford Nanopore sequencing currently has higher error rates than Illumina sequencing, although improvements in nanopore chemistry and basecalling could potentially reduce the differential [21]. The error rate of Oxford Nanopore long reads could also in part be compensated for by assembly algorithms through intensive computations to acquire a more accurate AMR profiling independent of other sequencing technologies such as Illumina sequencing. Therefore, we compared the AMR genotypes and phenotypes of selected bacterial pathogens, as predicted based on Oxford Nanopore long-read assemblies using different long-read assemblers.

Five genotypically antimicrobial-resistant strains with simulated reads were used (Supplementary Table S10). All Miniasm/Racon and Raven assemblies provided AMR profiles that were consistent with the reference genomes. Canu also produced assemblies that had accurate AMR profiles, with only one exception that the Canu assembly of *S. aureus* TW20 carried *erm(33)* that was absent in the reference genome. Both Flye and Shasta produced assemblies with inaccurate AMR profiles for three strains, while Redbean performed the worst and produced four Redbean assemblies with inconsistent AMR profiles with the reference genomes. Noticeably, when low-quality reads were used, no ARGs were identified in the Flye, Miniasm/Racon, and Redbean assemblies (Supplementary Table S11). Raven still produced the assembly of *Klebsiella variicola* DSM 15968 that had an accurate AMR profile, while the Raven assemblies of both *P. aeruginosa* PAO1 and *C. jejuni* NCTC 11168 also contained three ARGs that were present in the reference genomes.

Five genotypically antimicrobial-resistant strains with real reads were used (Supplementary Table S12). All assemblies of *P. aeruginosa* CFSAN084950 had consistent AMR profiles with the reference genome. The gene associated with kanamycin resistance, *aph(3′)-III*, was present in the Canu, Miniasm/Racon, Raven, and Redbean assemblies of *E. coli* O157:H7 CFSAN076619, while the gene associated with ampicillin resistance, *blaOXA-61*, was only identified in the Miniasm/Racon and Raven assemblies of *C. jejuni* NCTC 11168. Noticeably, none of the assemblies of *S. aureus* CFSAN007894 and *Campylobacter coli* CFSAN032805 carried the same ARGs with the reference genomes.

Overall, compared to other assemblers, Raven was more capable of generating assemblies for accurate AMR genotypes, closely followed by Miniasm/Racon, especially when low-quality reads were used. Although it is possible to assemble Oxford Nanopore long reads into complete genomes to help implement near real-time AMR profiling, doing so would compromise the genome accuracy of bacterial pathogens and result in inaccurate AMR genotypes. Future improvements to the library preparation and basecalling of Oxford Nanopore sequencing, as well as long-read assembly algorithms, may mitigate this deficiency.

### 2.3. Virulence Genes

Previous studies have demonstrated the effectiveness of Oxford Nanopore sequencing in the rapid identification of virulence genes in bacterial pathogens [22,23,24]. The numbers of virulence genes identified in all assemblies of mediocre-quality reads of each strain were not significantly different (*p* > 0.05) from those in the reference genome (Table 4). However, it should be noted that when low-quality reads were tested, only the Raven assemblies had numbers of virulence genes that were not significantly different (*p* > 0.05) from the reference genomes (Table 5). No significant differences (*p* > 0.05) were observed between the numbers of virulence genes of the Raven assemblies of mediocre- and low-quality reads, while the numbers of virulence genes of the Flye, Miniasm/Racon, and Redbean assemblies of low-quality reads became significantly lower (*p* < 0.05) compared to those of mediocre-quality reads. All assemblies of real reads of each strain had numbers of virulence genes not significantly different (*p* > 0.05) from those in the reference genome (Table 6). Taken together, all assemblers were able to generate assemblies containing accurate information about the virulence potential of bacterial pathogens, when Oxford Nanopore long reads with a lower level of errors were assembled. However, when more errors were introduced into Oxford Nanopore long reads, Raven was superior to other assemblers.

### 2.4. Multilocus Sequence Typing (MLST)

MLST has been considered as a portable approach to the identification of clones within populations of pathogens [25], thus enabling an exchange of molecular typing data for global epidemiology via the Internet. Tarumoto et al. [26] reported that the Canu assemblies of the Oxford Nanopore long reads of two *Enterococcus faecium* strains provided consistent MLST results with conventional Sanger sequencing, although a few mismatched bases on each locus were observed, especially in repeat sequences. In this study, we thus investigated which assemblers could best assemble Oxford Nanopore long reads for accurate MLST of bacterial pathogens.

It is worth noting that Miniasm/Racon was the most effective assembler for mediocre-quality reads (Table 7), whose assemblies contained the seven housekeeping genes for all strains tested. Canu and Raven also performed well for MLST, as only the Canu and Raven assemblies of *C. botulinum* CDC_1632 failed to have a positive MLST result. The Flye assemblies showed positive MLST results only for three strains, while the Redbean assemblies of up to eight strains had negative MLST results. Shasta performed the worst and did not generate any assemblies with positive MLST results. Noticeably, when the assemblies of low-quality reads were evaluated for MLST (Table 8), the Raven assemblies of *E. coli* O157:H7 Sakai and *K. variicola* DSM 15968 showed positive MLST results among all strains tested.

Concerning real reads, Miniasm/Racon and Raven performed equally well (Table 9), as their assemblies did not harbor all seven housekeeping genes only for *C. botulinum* CFSAN034200. Canu and Redbean produced assemblies of *C. coli* CFSAN032805 and *C. jejuni* NCTC 11168 with negative MLST results. In contrast, there were six Shasta assemblies with positive MLST results, while only the Flye assemblies of *P. aeruginosa* CFSAN084950 and *C. sakazakii* CFSAN068773 had positive MLST results.

### 2.5. Whole-Genome Phylogeny

**Figure 1:**
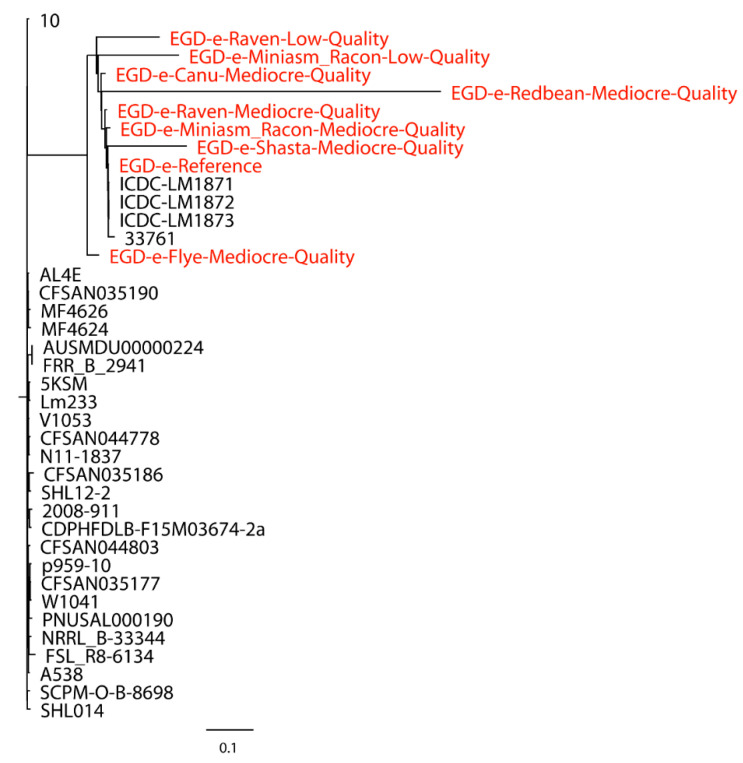
Whole-genome phylogenetic tree of Oxford Nanopore long-read assemblies of *Listeria monocytogenes* EGD-e with mediocre- and low-quality reads using different long-read assemblers, as aligned to 30 distantly related *L. monocytogenes* strains selected based on the single-nucleotide polymorphisms (SNPs) strategy (number of SNPs > 500) and compared with the reference genome.

**Figure 2:**
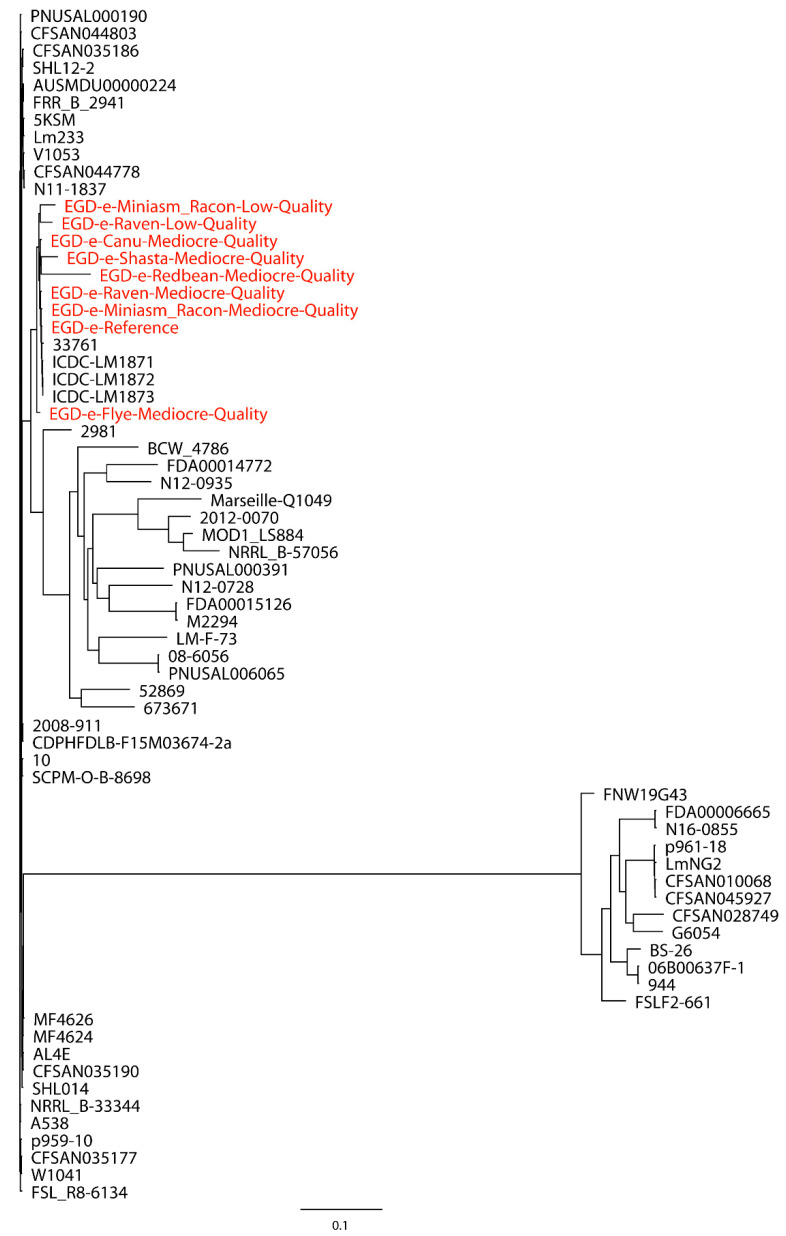
Whole-genome phylogenetic tree of Oxford Nanopore long-read assemblies of *Listeria monocytogenes* EGD-e with mediocre- and low-quality reads using different long-read assemblers, as aligned to 30 distantly related *L. monocytogenes* strains selected based on the single-nucleotide polymorphisms (SNPs) strategy (number of SNPs > 500) and 30 closely related *L. monocytogenes* strains selected based on the SNP (number of SNPs < 500) and core-genome multilocus sequence typing (cgMLST) (different alleles < 500) strategies and compared with the reference genome.

Taylor et al. [27] used 23 closely related *Salmonella* strains (number of SNPs < 500) to build a whole-genome phylogenetic tree with the Miniasm/Racon assembly of the Oxford Nanopore long reads of *S.* Bareilly CFSAN000189, where they observed a congruent topology between trees built with the SPAdes assembly of Illumina short reads and the Miniasm/Racon assembly. In our work, both closely and distantly related strains were included when evaluating the performance of Oxford Nanopore long-read assemblies in the phylogenetic analyses. A total of 30 closely related *L. monocytogenes* strains were first used for the phylogenetic analysis of *L. monocytogenes* EGD-e with mediocre- and low-quality reads. The Redbean assembly of low-quality reads was not included due to the error produced by CSI Phylogeny when this assembly with an inaccurate genome size of 30,776 bp was processed. As shown in Figure 1, the assemblies formed a single monophyletic clade with the reference genome and closely related strains, irrespective of the assemblies of mediocre- or low-quality reads, although the Flye assembly of mediocre-quality reads formed a dispersed paraphyletic clade relative to the major clade of the reference genome and other assemblies. The Miniasm/Racon and Raven assemblies of mediocre-quality reads had the smallest distance from the reference genome. Interestingly, the Miniasm/Racon assembly of low-quality reads with an inaccurate genome size of 542,915 bp was also on the clade where the reference genome was located. When 30 distantly related *L. monocytogenes* strains were also included in the phylogenetic analysis of *L. monocytogenes* EGD-e, a similar clade topology of the monophyletic clade that included the reference genome and assemblies were observed (Figure 2), where the Miniasm/Racon and Raven assemblies still had the smallest distance from the reference genome.

**Figure 3:**
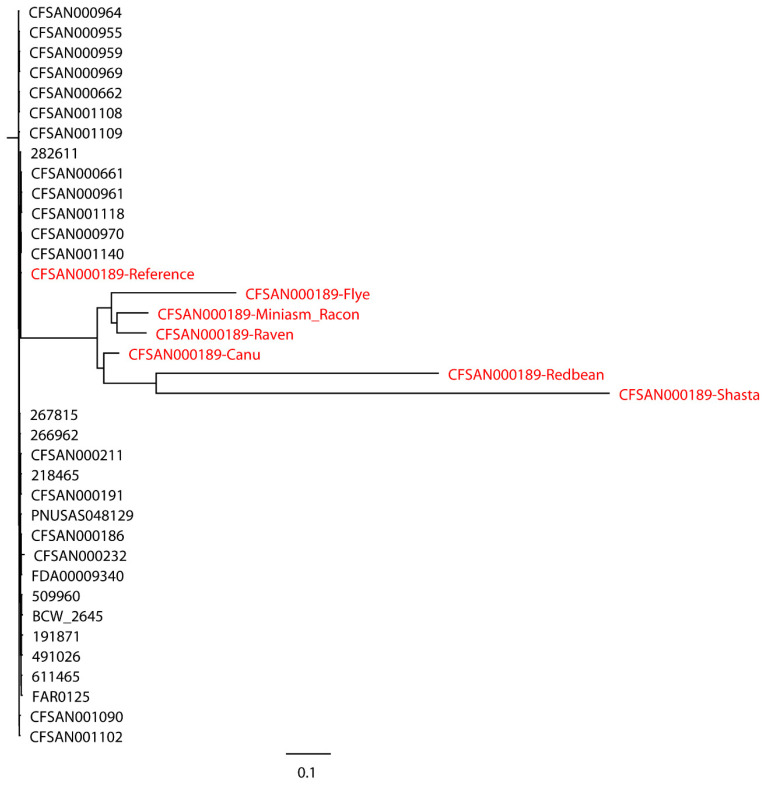
Whole-genome phylogenetic tree of Oxford Nanopore long-read assemblies of *Salmonella* Bareilly CFSAN000189 with real reads using different long-read assemblers, as aligned to 30 closely related *S.* Bareilly strains selected based on the single-nucleotide polymorphisms (SNPs) (number of SNPs < 500) and whole-genome multilocus sequence typing (wgMLST) strategies (different alleles < 500) and compared with the reference genome.

**Figure 4:**
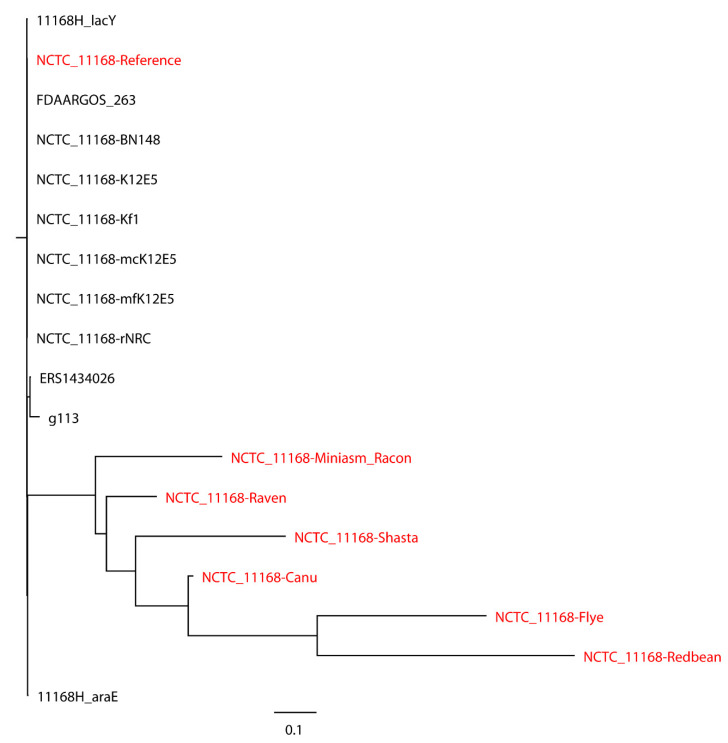
Whole-genome phylogenetic tree of Oxford Nanopore long-read assemblies of *Campylobacter jejuni* NCTC 11168 with real reads using different long-read assemblers, as aligned to 11 closely related *C. jejuni* strains selected based on the single-nucleotide polymorphisms (SNPs) strategy (number of SNPs < 500) and compared with the reference genome.

**Figure 5:**
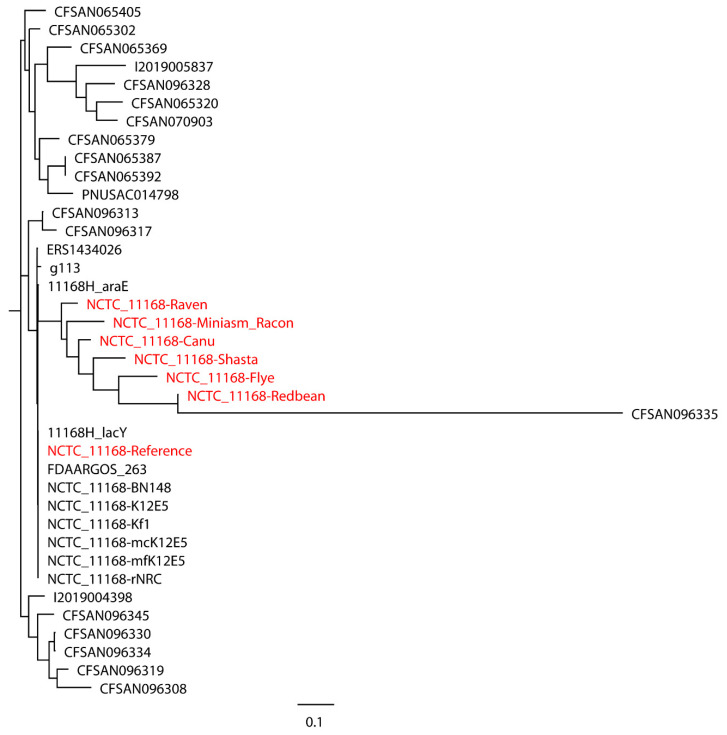
Whole-genome phylogenetic tree of Oxford nanopore long-read assemblies of *Campylobacter jejuni* NCTC 11168 with real reads using different long-read assemblers, as aligned to 11 closely related (number of SNPs < 500) and 20 distantly related *C. jejuni* strains (number of SNPs > 500) selected based on the single-nucleotide polymorphisms (SNPs) strategy and compared with the reference genome.

**Figure 6:**
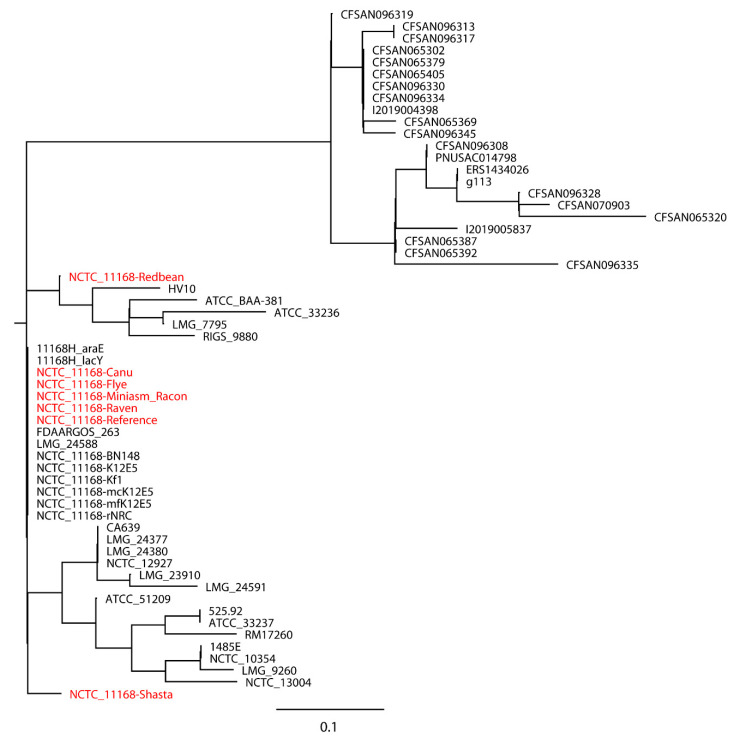
Whole-genome phylogenetic tree of Oxford nanopore long-read assemblies of *Campylobacter jejuni* NCTC 11168 with real reads using different long-read assemblers, as aligned to 11 closely related (number of SNPs < 500), 20 distantly related *C. jejuni* strains (number of SNPs > 500) selected based on the single-nucleotide polymorphisms (SNPs) strategy, and 20 *Campylobacter* strains of other species and compared with the reference genome.

*S.* Bareilly CFSAN000189 assemblies with real reads and its reference genome were aligned to 30 closely related *S.* Bareilly strains (Figure 3). All 30 closely related strains and the reference genome formed a single monophyletic clade, whereas the assemblies formed a dispersed paraphyletic group sister to the reference genome clade. Similar results were obtained for the tree of *C. jejuni* NCTC 11168 with real reads built with 11 closely related *C. jejuni* strains selected based on the SNP strategy (Figure 4), where the reference genome formed a monophyletic group with the closely related strains and the assemblies were a non-monophyletic group forming multiple independent clades. When 20 distantly related *C. jejuni* strains were included in the phylogenetic analysis of *C. jejuni* NCTC 11168, the assemblies were still placed on different clades compared to the reference genome (Figure 5). Although biased results were observed across all assemblies of both *S.* Bareilly CFSAN000189 and *C. jejuni* NCTC 11168, the Miniasm/Racon and Raven assemblies always had the smallest distance from the reference genomes. We then included 20 *Campylobacter* strains of other species in the phylogenetic analysis of *C. jejuni* NCTC 11168 (Figure 6). Interestingly, all assemblies except the Redbean and Shasta assemblies were on the clade where the reference genome was located. The Miniasm/Racon and Raven assemblies still displayed the smallest distance from the reference genome.

The higher error rates of Oxford Nanopore long reads may confound the phylogenetic differences between the assemblies and closely related strains selected based on the SNP and core-genome MLST (cgMLST) or whole-genome MLST (wgMLST) strategies, further confirming our observations that Oxford Nanopore long-read assemblies contained SNPs (Supplementary Tables S2, S4 and S6) and also produced inaccurate MLST results (Table 9, Table 10 and Table 11) relative to the reference genomes. We identified some recurrent phylogenetic patterns of long-read assemblies that could potentially be addressed in the future, as continued improvements in nanopore chemistry and basecalling would systematically mitigate the high numbers of errors to achieve a more accurate phylogenetic inference.

### 2.6. Pan Genomes

**Figure 7:**
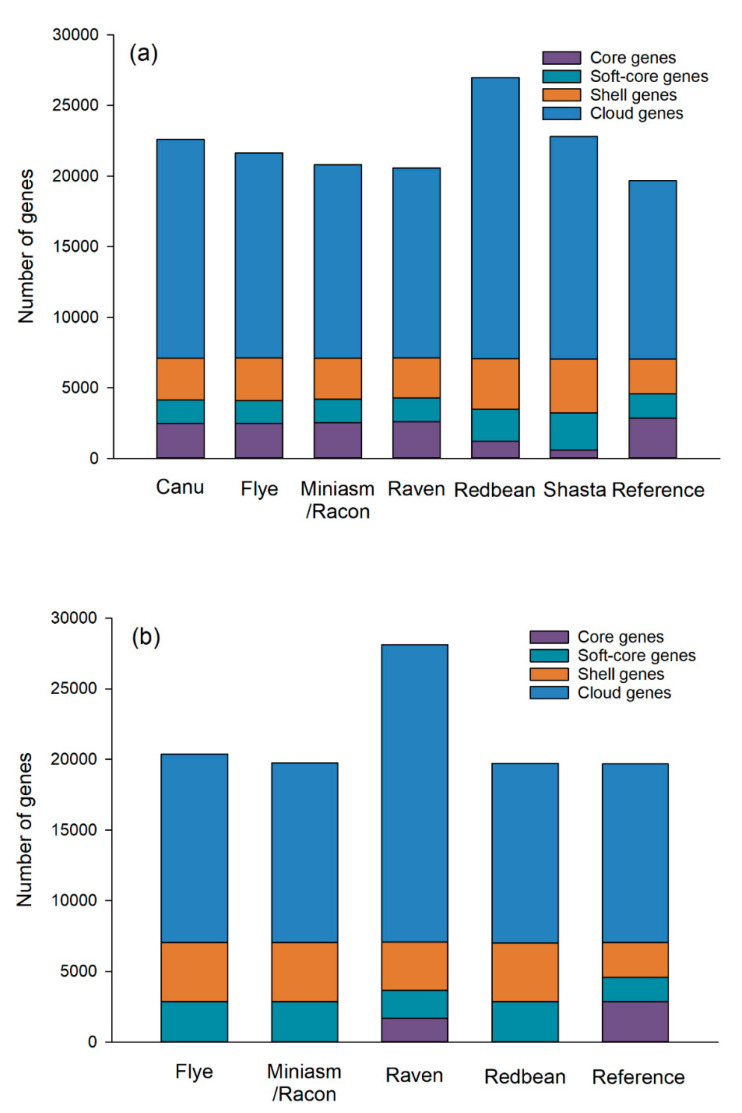
Pan genomes of Oxford Nanopore long-read assemblies of *Pseudomonas aeruginosa* PAO1 with mediocre- (**a**) and low-quality (**b**) reads using different long-read assemblers and 20 distantly related *P. aeruginosa* strains selected based on the single-nucleotide polymorphisms (SNPs) strategy (number of SNPs > 500) and compared with the reference genome.

A total of 20 distantly related *P. aeruginosa* strains were included in the pan-genome analysis of *P. aeruginosa* PAO1 with mediocre- and low-quality reads. Raven was the most effective assembler for the pan-genome analysis of *P. aeruginosa* PAO1 with mediocre-quality reads, which displayed the numbers of core (core and soft-core) and accessory (shell and cloud) genes that were most similar to the reference genome (Figure 7). The pan genomes of the Raven assembly consisted of a total of 20,583 genes with 2615 core genes (12.7%) and 17,968 accessory genes (87.3%). Similarly, there were a total of 19,673 genes with 2866 core genes (14.6%) and 16,807 accessory genes (85.4%) in the pan genomes of the reference genome. Our results thus demonstrate that Raven could tolerate the inaccuracy of Oxford Nanopore long reads. Raven was closely followed by Miniasm/Racon that was also able to achieve similar pan-genome patterns with the reference genome. Canu, Flye, and Shasta were moderate performers for the pan-genome analysis, while Redbean was the least effective, producing inaccurate numbers of core genes (4.6%) and accessory genes (95.4%), respectively.

Regarding the pan-genome analysis of the assemblies of low-quality reads, the Flye, Miniasm/Racon, and Redbean assemblies did not produce accurate numbers of core genes, although their total numbers of genes were similar to the reference genome, which could be attributed to their inaccurate genome sizes. For the pan genome of the Raven assembly of low-quality reads, we observed a dramatic increase in the total number of genes (28,119) compared to that of mediocre-quality reads, with a decrease in the number of core genes (1696) and an increase in the number of accessory genes (26,423).

**Figure 8:**
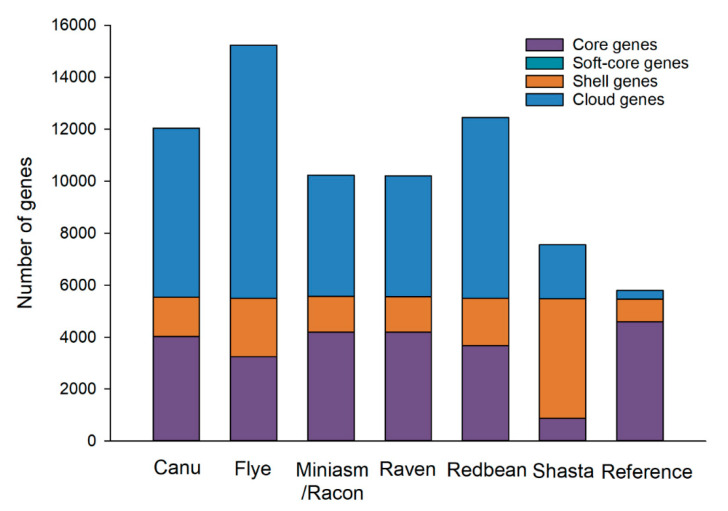
Pan genomes of Oxford Nanopore long-read assemblies of *Escherichia coli* O157:H7 CFSAN076619 with real reads using different long-read assemblers and six closely related *E. coli* O157:H7 strains selected based on the whole-genome multilocus sequence typing (wgMLST) strategy (different alleles < 500) and compared with the reference genome.

Six closely related strains of *E. coli* O157:H7 CFSAN076619 with real reads were found based on the SNP and wgMLST strategies, which were thus included in its pan-genome analysis. The pan genome of the reference genome and the six strains consisted of 5790 genes with 4595 core genes (79.4%) and 1195 accessory genes (20.6%) (Figure 8). The Miniasm/Racon and Raven assemblies had numbers of core genes most similar to the reference genome compared to other assemblies, although the pan genomes of all assemblies contained large numbers of accessory genes. One key reason for this poor performance is the inherently limited accuracy of Oxford Nanopore long reads, as also revealed by our SNP and indel analyses, demonstrating that the degree of errors in an assembly can greatly affect conclusions about gene presence and absence beyond just the inability to resolve genomic structures. SNPs and indels detected in the assemblies of Oxford Nanopore long reads could introduce truncated genes that can produce a large number of misannotated genes, which increased the numbers of accessory genes in the pan genomes [18]. Our pan-genome analyses likely reflect the difficulty in using highly error-prone Oxford Nanopore long reads for accurately annotating genes due to gene truncation and misplaced start sites. Another aspect worth noting is that this limitation of Oxford Nanopore sequencing could become more remarkable when closely related strains were included in the pan-genome analysis. Our pan-genome analyses thus again highlight the limitation of Oxford Nanopore sequencing to produce accurate long reads. Overcoming this existing deficiency could greatly boost the annotations of many clinically and microbiologically important genomic regions in bacterial pathogens. Despite all these observations using real reads, Miniasm/Racon and Raven were comparable for the pan-genome analysis and performed the best among all assemblers tested, although both of their pan genomes still contained over 6000 accessory genes.

### 2.7. Raven and Miniasm/Racon

Based on the performance in various genomic analyses, overall, the most robust and accurate long-read assembler was Raven, closely followed by Miniasm/Racon. Interestingly, Raven is built upon modules of Miniasm and Racon. However, Raven executes novel algorithms for the overlap and layout phases of genome assembly that increase the contiguity of the final assembly [14]. Miniasm provides an ultrafast *de novo* assembly for long noisy reads, although it does not have a consensus step [12]. While the lack of error correction results in assemblies with a significant error rate, it can be enhanced with a suitably efficient consensus phase using an additional polishing step [28,29]. Racon contains a standalone consensus module for raw uncorrected assemblies of long reads generated by rapid assembly methods that do not include a consensus step [13]. Senol Cali et al. [30] conducted a review to analyze state-of-the-art tools using Oxford Nanopore long reads in terms of accuracy, speed, memory efficiency, and scalability. After a comprehensive analysis, they recommended that Miniasm and Racon should be used for assembly and polishing, respectively. Our findings further demonstrate the effectiveness of Miniasm/Racon-based assembly processes for Oxford Nanopore long reads, suggesting that assembly strategies following that algorithmic model may be most fruitful in the future. We acknowledge that not all currently available assemblers were included because the field of long-read assembly continues to evolve. The assemblers chosen for benchmarking have been widely used in the published literature of genome assembly beyond the initial release of the software, which also differ in their underlying assembly algorithms. As such the comparisons were more associated with their algorithms. We chose to benchmark the long-read assemblers with their default parameters and recommended settings. Future optimization of these parameters and settings before implementation could potentially improve their assembly algorithms. We can anticipate that with further development of Oxford Nanopore sequencing technology, read quality will increase, which could make assembly using Oxford Nanopore long reads more accurate and applicable to a wide range of bacterial pathogens.

## 3. Materials and Methods

### 3.1. Simulated Oxford Nanopore Long Reads

To assess whether long-read assemblers could tolerate problems encountered in real error-prone Oxford Nanopore long reads, the complete genomes of 10 species (10 strains) of bacterial pathogens, spanning a wide range of genome sizes and GC contents, were selected from the National Center for Biotechnology Information (NCBI) (Table 10), which were used as the reference genomes in our study.

Badread 0.1.5 [31] was used based on the Nanopore error model to mimic simulated Oxford Nanopore long reads of mediocre quality, with a mean fragment size of 15,000 bp, fragment size standard deviation of 13,000 bp, mean identity of 85, max identity of 95, identity standard deviation of 5, and coverage of 50×. The chimera join rate, junk read rate, and random read rate of each simulated mediocre-quality dataset were adjusted to 1%. Oxford Nanopore long reads of low quality were also simulated using Badread by artificially introducing more chimeras, low-quality regions, and systematic basecalling errors, with a glitch rate of 1000, glitch size of 100, glitch skip of 100, mean identity of 75, max identity of 90, identity standard deviation of 8, and coverage of 50×. The chimera join rate, junk read rate, and random read rate of each simulated low-quality dataset were adjusted to 10%, 5%, and 5%, respectively. Oxford Nanopore ligation adapters were added to the start and end of each read using Badread, with a start adapter rate of 90 and start adapter amount of 60, and an end adapter rate of 50 and end adapter amount of 20. Start and end adapter sequences were AATGTACTTCGTTCAGTTACGTATTGCT and GCAATACGTAACTGAACGAAGT, respectively [31].

### 3.2. Real Oxford Nanopore Long Reads

Ten species (10 strains) of bacterial pathogens with publicly available complete genomes from the NCBI were tested for real Oxford Nanopore long reads, together covering a wide range of genome sizes and GC contents (Table 11). The complete genomes were used as the reference genomes in this study. Oxford Nanopore long reads were downloaded from the Sequence Read Archive (SRA) of the NCBI.

### 3.3. Long-Read Assemblers

We assembled Oxford Nanopore long reads using six long-read assemblers with default parameters, namely Canu 1.8, Flye 2.3.7, Miniasm 0.2/Racon 1.3.1.1, Raven 0.0.8, Redbean 2.0, and Shasta 0.4.0. Miniasm/Racon was applied with two rounds of Racon polishing after the initial Miniasm assembly. Canu, Flye, and Redbean require a specified genome size as input. Flye, Miniasm/Racon, and Shasta indicate circularity by producing GFA files of their final assemblies, while Canu signals circularity via ‘suggestCircular’ in the header lines of FASTA files. However, Raven and Redbean do not indicate whether a contig is circular or not.

### 3.4. Computational Environments

Assemblies with Raven and Shasta were carried out on the Linux operating system of Ubuntu 18.04.4 LTS on a computer with 16 threads of CPU and 32 GB of RAM. Twelve threads of CPU were allocated to each assembler in the option of the number of threads used. Canu, Flye, Miniasm/Racon, and Redbean were available on the Amazon Web Services (AWS)-based GalaxyTrakr platform developed by the U.S. Food and Drug Administration (FDA) and intended for use by GenomeTrakr laboratories [32].

### 3.5. Assessment of Genome Completeness and Accuracy

Assembly quality was evaluated using Quast 5.0.2 [33] by computing several metrics, including the number of contigs, total length (bp), GC content, and indels. The number of indels was reported as the number of indels per one million bp of the reference genome. BUSCO 4.0.6 [34] was used to provide a quantitative assessment of genome completeness, with 0.01 as the E-value cutoff for BLAST searches and three candidate regions to consider. The results were expressed as complete BUSCOs that represent the fraction of the expected gene complement with full-length reading frames. CSI Phylogeny 1.4 [35] was used to call SNPs of each long-read assembly relative to the corresponding reference genome. Default settings were used, with 10× as the minimum depth at SNP positions, 10% as the minimum relative depth at SNP positions, 10 bp as the minimum distance between SNPs, 30 as the minimum SNP quality, 25 as the minimum read mapping quality, and 1.96 as the minimum Z-score. The reference genome of each strain was used for SNP calling. The number of SNPs was expressed as the number of SNPs per one million bp of the reference genome.

### 3.6. Identifications of Plasmids, ARGs, and Virulence Genes

Plasmids were identified and typed using staramr 0.6.0 (https://github.com/phac-nml/staramr) against known plasmid sequences in the PlasmidFinder database [36] with 98% minimum identity and 60% minimum coverage. Mass screening of ARGs was conducted using staramr against known gene sequences in the ResFinder database [37] with a 98% minimum identity and 60% minimum coverage and the PointFinder database [38] with 98% minimum identity and 95% minimum coverage, respectively. Virulence genes were detected using ABRicate 0.8.7 (https://github.com/tseemanN.A.bricate) integrated with the Virulence Factors Database (VFDB) for bacterial pathogens [39] with 80% minimum identity and 60% minimum coverage compared with known gene sequences.

### 3.7. MLST

MLST was performed with mlst 2.19.0 (https://github.com/tseemann/mlst) that incorporates the components of the PubMLST database [40] through comparing assemblies against traditional PubMLST typing schemes based on seven housekeeping genes using default settings, with 95% as the minimum identity of full allele, 10% as the minimum coverage of partial allele, and 50 as the minimum score to match a scheme.

### 3.8. Whole-Genome Phylogenetic Analyses

*Listeria monocytogenes* EGD-e was used for the phylogenetic analysis of the assemblies of simulated reads. We first selected 30 closely related *L. monocytogenes* strains (Supplementary Table S13) based on the SNP (number of SNPs < 500) and cgMLST (different alleles < 500) strategies using BacWGSTdb 2.0 [41]. We then added 30 distantly related *L. monocytogenes* strains (Supplementary Table S14) selected based on the SNP strategy (number of SNPs > 500) using BacWGSTdb to the 30 closely related *L. monocytogenes* strains.

*Salmonella* Bareilly CFSAN000189 and *Campylobacter jejuni* NCTC 11168 were used for the phylogenetic analysis of the assemblies of real reads. To perform the phylogenetic analysis, long-read assemblies of these two strains were aligned to datasets of 30 closely related *S.* Bareilly (Supplementary Table S15) and 11 closely related *C. jejuni* strains (Supplementary Table S16), respectively. The closely related datasets were selected based on the SNP (number of SNPs < 500) and wgMLST strategies (different alleles < 500) using BacWGSTdb. Twenty distantly related *C. jejuni* strains (number of SNPs > 500) and 20 *Campylobacter* strains of other species were also included in the phylogenetic analysis of *C. jejuni* NCTC 11168 (Supplementary Table S17).

The whole-genome phylogenetic analyses were performed with all long-read assemblies and their corresponding reference genomes, as aligned to the selected datasets. CSI Phylogeny was used with default settings as previously described to call SNPs of long-read assemblies and to calculate phylogenetic relationships based on the concatenated alignment of the high-quality SNPs. *L. monocytogenes* AT3E (RefSeq assembly accession: GCF_002557735.1), *S. typhimurium* LT2 (RefSeq assembly accession: GCF_000006945.2), and *C. jejuni* isolate_W1 (RefSeq assembly accession: GCF_002179165.1) served as the reference genomes for *L. monocytogenes* EGD-e, *S.* Bareilly CFSAN000189, and *C. jejuni* NCTC 11168, respectively. The inferred maximum-likelihood whole-genome phylogeny was visualized as a cladogram with Geneious Prime 2020.1.2. (Biomatters, Ltd., Auckland, New Zealand).

### 3.9. Pan-Genome Analyses

To conduct the pan-genome analyses, genome sequences were first annotated using Prokka 1.14.0 [42]. Afterward, pan genomes were then analyzed with Roary 3.12.0 [43] by using genome annotations from Prokka to calculate the numbers of core, soft-core, shell, and cloud genes that are found in >99%, 95–99%, 15–95%, and <15% of genomes, respectively.

*Pseudomonas aeruginosa* PAO1 and *Escherichia coli* O157:H7 CFSAN076619 were used for the pan-genome analyses of simulated and real reads, respectively. Thirty distantly related *P. aeruginosa* PAO1 (Supplementary Table S18) were used for the pan-genome analysis of *P. aeruginosa* PAO1. For the pan-genome analysis of *E. coli* O157:H7 CFSAN076619, six closely related *E. coli* O157:H7 strains (Supplementary Table S19) were selected based on the SNP and wgMLST strategies using BacWGSTdb.

### 3.10. Statistical Analyses

The Wilcoxon signed-rank test was performed using SigmaPlot 14.0 (Systat Software Inc., San Jose, CA, USA) to determine whether significant differences (*p* < 0.05) existed between the reference genomes and completed long-read assemblies in genome size, GC content, complete BUSCOs, and the number of virulence genes, among completed long-read assemblies in numbers of SNPs and indels. This test was also conducted to determine whether there were significant differences (*p* < 0.05) between the assemblies of mediocre- and low-quality reads in genome size, GC content, complete BUSCOs, and the number of virulence genes, SNPs, and indels.

## 4. Conclusions

The significance of long-read assembly will continue to grow as Oxford Nanopore sequencing is becoming more and more widely used in the genomics of bacterial pathogens. An ideal long-read assembler for bacterial pathogens should efficiently complete the assembly process and remain robust against a wide range of systematic error-prone long reads. Most importantly, the genome assembly generated should be able to provide accurate genomic information for downstream pathogen identification, genotypic prediction, genome-wide annotation, and phylogenetic inference.

Our benchmarking using both simulated (mediocre- and low-quality) and real reads highlights the salient fact that Oxford Nanopore sequencing errors may cause difficulties in resolving the phylogenetic differences in some cases. Similarly, our pan-genome analyses also demonstrate the limitation of using error-prone Oxford Nanopore long reads to produce a high-quality assembly for accurate genome annotation. These problems could be addressed with the improvement of nanopore chemistry and basecalling. Overall, the most robust and accurate long-read assembler was Raven, closely followed by Miniasm/Racon, as revealed by its performance in various genomic analyses, although Raven does not indicate whether a contig is circular or not. With the improvement of sequencing technologies and assembly algorithms, defining an optimal long-read assembly approach for genomic analyses of bacterial pathogens is a continuous process.

To decrease the assembly errors caused by a single assembler to the desired level, it is warranted to rely on multiple assemblers to perform standalone assemblies and compare their independent results in genomic analyses. This could provide the opportunity for additional data validation, as confidence in one assembly becomes greater only when its results are consistent with those of other assemblies.

## Figures and Tables

**Table 1:** Oxford Nanopore long-read assemblies of bacterial strains with mediocre-quality reads using different long-read assemblers compared to their corresponding reference genomes.

Assembly Properties	Assembler	*Pseudomonas aeruginosa* PAO1	*Escherichia coli* O157:H7 Sakai	*Bacillus anthracis* Ames Ancestor	*Klebsiella variicola* DSM 15968	*Salmonella* Typhimurium LT2	*Cronobacter sakazakii* ATCC 29544	*Clostridium botulinum* CDC_1632	*Listeria monocytogenes* EGD-e	*Staphylococcus aureus* TW20	*Campylobacter jejuni* NCTC 11168
Number of contigs	Canu	4 (0 cir.^a^)	4 (0 cir.)	4 (0 cir.)	2 (0 cir.)	5 (0 cir.)	4 (0 cir.)	1 (0 cir.)	1 (0 cir.)	1 (0 cir.)	1 (0 cir.)
	Flye	5 (3 cir.)	3 (1 cir.)	5 (2 cir.)	3 (2 cir.)	6 (4 cir.)	5 (1 cir.)	3 (2 cir.)	1 (0 cir.)	3 (0 cir.)	1 (0 cir.)
	Miniasm/Racon	1 (0 cir.)	1 (0 cir.)	4 (0 cir.)	1 (0 cir.)	1 (0 cir.)	3 (0 cir.)	1 (0 cir.)	1 (0 cir.)	1 (0 cir.)	1 (0 cir.)
	Raven	1 (N.A.)	1 (N.A.)	3 (N.A.)	1 (N.A.)	1 (N.A.)	1 (N.A.)	1 (N.A.)	1 (N.A.)	1 (N.A.)	1 (N.A.)
	Redbean	17 (N.A.)	29 (N.A.)	17 (N.A.)	15 (N.A.)	18 (N.A.)	11 (N.A.)	12 (N.A.)	11 (N.A.)	10 (N.A.)	6 (N.A.)
	Shasta	6 (0 cir.)	12 (0 cir.)	9 (0 cir.)	2 (0 cir.)	7 (0 cir.)	6 (0 cir.)	1 (0 cir.)	2 (0 cir.)	3 (0 cir.)	1 (0 cir.)
	Reference	1 (1 cir.)	3 (3 cir.)	3 (3 cir.)	1 (1 cir.)	2 (2 cir.)	4 (4 cir.)	1 (1 cir.)	1 (1 cir.)	3 (3 cir.)	1 (1 cir.)
Total length (bp)	Canu	6,333,544	5,568,563	5,499,817	5,498,961	4,979,769	4,591,713	4,354,206	2,925,837	3,011,570	1,624,925
	Flye	6,321,807	5,609,732	5,554,459	5,540,113	4,977,099	4,688,438	4,440,030	2,973,099	3,093,259	1,669,691
	Miniasm/Racon	6,261,224	5,476,636	5,417,911	5,454,862	4,852,014	4,667,511	4,382,592	2,930,573	3,000,850	1,580,648
	Raven	6,260,945	5,492,769	5,494,259	5,515,533	4,852,094	4,507,398	4,383,585	2,939,555	3,038,812	1,637,228
	Redbean	6,328,846	5,622,673	5,569,009	5,568,381	5,077,817	4,643,262	4,426,510	3,033,177	3,139,954	1,653,174
	Shasta	6,211,462	5,341,056	5,443,627	5,505,281	4,873,783	4,594,622	4,379,095	2,922,932	3,050,977	1,629,400
	Reference	6,264,404	5,594,605	5,503,926	5,521,203	4,951,383	4,663,565	4,393,047	2,944,528	3,075,806	1,641,481
GC content (%)	Canu	66.80	50.52	35.31	57.56	51.84	56.74	28.13	38.11	32.87	30.76
	Flye	66.55	50.29	34.94	57.40	52.04	56.46	27.68	37.63	32.48	30.04
	Miniasm/Racon	66.55	50.54	35.23	57.54	52.23	56.79	28.04	38.02	32.79	30.54
	Raven	66.55	50.55	35.27	57.56	52.23	56.72	28.05	38.01	32.81	30.60
	Redbean	65.99	50.42	35.65	57.22	52.06	56.44	28.58	38.40	33.39	31.10
	Shasta	66.52	50.50	35.31	57.54	52.26	56.66	28.09	38.03	32.88	30.60
	Reference	66.56	50.48	35.24	57.56	52.24	56.64	28.02	37.98	32.78	30.55

^a^ cir., circularized contigs. All circularized contigs were plasmids and the chromosomes were not circularized.

**Table 2:** Oxford Nanopore long-read assemblies of bacterial strains with low-quality reads using different long-read assemblers compared to their corresponding reference genomes.

Assembly Properties	Assembler	*Pseudomonas aeruginosa* PAO1	*Escherichia coli* O157:H7 Sakai	*Bacillus anthracis* Ames Ancestor	*Klebsiella variicola* DSM 15968	*Salmonella* Typhimurium LT2	*Cronobacter sakazakii* ATCC 29544	*Clostridium botulinum* CDC_1632	*Listeria monocytogenes* EGD-e	*Staphylococcus aureus* TW20	*Campylobacter jejuni* NCTC 11168
Number of contigs	Canu	N.C.^a^	N.C.	N.C.	N.C.	N.C.	N.C.	N.C.	N.C.	N.C.	N.C.
	Flye	21 (1 cir.^b^)	N.C.	15 (1 cir.)	13 (0 cir.)	12 (1 cir.)	15 (4 cir.)	14 (1 cir.)	N.C.	8 (1 cir.)	N.C.
	Miniasm/Racon	6 (0 cir.)	14 (0 cir.)	18 (0 cir.)	6 (0 cir.)	12 (0 cir.)	7 (0 cir.)	15 (1 cir.)	10 (0 cir.)	15 (0 cir.)	6 (0 cir.)
	Raven	2 (N.A.^c^)	5 (N.A.)	5 (N.A.)	5 (N.A.)	3 (N.A.)	1 (N.A.)	4 (N.A.)	3 (N.A.)	2 (N.A.)	1 (N.A.)
	Redbean	5 (N.A.)	3 (N.A.)	1 (N.A.)	2 (N.A.)	5 (N.A.)	1 (N.A.)	3 (N.A.)	3 (N.A.)	3 (N.A.)	N.C.
	Shasta	N.C.	N.C.	N.C.	N.C.	N.C.	N.C.	N.C.	N.C.	N.C.	N.C.
	Reference	1 (1 cir.)	3 (3 cir.)	3 (3 cir.)	1 (1 cir.)	2 (2 cir.)	4 (4 cir.)	1 (1 cir.)	1 (1 cir.)	3 (3 cir.)	1 (1 cir.)
Total length (bp)	Canu	N.C.	N.C.	N.C.	N.C.	N.C.	N.C.	N.C.	N.C.	N.C.	N.C.
	Flye	736,936	N.C.	525,387	351,544	404,682	431,809	494,295	N.C.	239,757	N.C.
	Miniasm/Racon	253,823	679,130	981,418	311,527	799,626	327,538	892,442	542,915	902,901	392,860
	Raven	6,320,253	5,678,114	5,351,012	5,671,912	4,902,124	4,508,440	4,419,225	2,950,805	3,036,007	1,625,458
	Redbean	67,981	57,373	5,507	25,385	36,862	4850	31,489	30,776	51,207	N.C.
	Shasta	N.C.	N.C.	N.C.	N.C.	N.C.	N.C.	N.C.	N.C.	N.C.	N.C.
	Reference	6,264,404	5,594,605	5,503,926	5,521,203	4,951,383	4,663,565	4,393,047	2,944,528	3,075,806	1,641,481
GC content (%)	Canu	N.C.	N.C.	N.C.	N.C.	N.C.	N.C.	N.C.	N.C.	N.C.	N.C.
	Flye	54.20	N.C.	45.56	53.62	44.26	54.74	51.02	N.C.	53.95	N.C.
	Miniasm/Racon	76.90	50.62	32.91	57.77	51.69	56.45	24.55	37.46	31.49	26.75
	Raven	66.45	50.41	35.19	57.39	52.17	56.65	27.96	37.98	32.74	30.59
	Redbean	52.89	50.55	33.36	59.61	45.32	54.91	32.18	28.09	35.27	N.C.
	Shasta	N.C.	N.C.	N.C.	N.C.	N.C.	N.C.	N.C.	N.C.	N.C.	N.C.
	Reference	66.56	50.48	35.24	57.56	52.24	56.64	28.02	37.98	32.78	30.55

^a^ N.C., not completed. ^b^ cir., circularized contigs. All circularized contigs were plasmids and the chromosomes were not circularized. ^c^ N.A., not applicable.

**Table 3:** Oxford Nanopore long-read assemblies of bacterial strains with real reads using different long-read assemblers compared to their corresponding reference genomes.

Assembly Properties	Assembler	*Pseudomonas aeruginosa* CFSAN084950	*Bacillus paranthracis* CFSAN068816	*Escherichia coli* O157:H7 CFSAN076619	*Salmonella* Bareilly CFSAN000189	*Cronobacter sakazakii* CFSAN068773	*Clostridium botulinum* CFSAN034200	*Listeria monocytogenes* CFSAN023468	*Staphylococcus aureus* CFSAN007894	*Campylobacter coli* CFSAN032805	*Campylobacter jejuni* NCTC 11168
Number of contigs	Canu	1 (1 cir.^a^)	4 (1 cir.)	3 (1 cir.)	4 (1 cir.)	9 (3 cir.)	4 (2 cir.)	1 (0 cir.)	2 (1 cir.)	3 (2 cir.)	1 (1 cir.)
	Flye	1 (1 cir.)	4 (4 cir.)	2 (2 cir.)	2 (1 cir.)	3 (3 cir.)	3 (3 cir.)	1 (1 cir.)	2 (2 cir.)	3 (3 cir.)	1 (1 cir.)
	Miniasm/Racon	1 (0 cir.)	6 (2 cir.)	2 (1 cir.)	2 (2 cir.)	7 (7 cir.)	227 (0 cir.)	1 (1 cir.)	3 (1 cir.)	3 (3 cir.)	43 (2 cir.)
	Raven	1 (N.A.^b^)	4 (N.A.)	2 (N.A.)	2 (N.A.)	1 (N.A.)	3 (N.A.)	1 (N.A.)	3 (N.A.)	3 (N.A.)	11 (N.A.)
	Redbean	6 (N.A.)	40 (N.A.)	28 (N.A.)	93 (N.A.)	17 (N.A.)	103 (N.A.)	14 (N.A.)	7 (N.A.)	2 (N.A.)	100 (N.A.)
	Shasta	1 (1 cir.)	4 (2 cir.)	10 (1 cir.)	2 (2 cir.)	850 (N.A.)	3 (3 cir.)	1 (1 cir.)	99 (N.A.)	10 (2 cir.)	1 (1 cir.)
	Reference	1 (1 cir.)	6 (6 cir.)	3 (3 cir.)	2 (2 cir.)	4 (4 cir.)	3 (3 cir.)	1 (1 cir.)	2 (2 cir.)	3 (3 cir.)	1 (1 cir.)
Total length (bp)	Canu	6,436,884	5,653,362	5,556,020	4,881,447	4,686,504	4,224,595	2,910,505	2,881,175	1,791,736	1,634,857
	Flye	6,462,251	5,761,528	5,499,303	4,862,479	4,659,214	4,294,319	3,008,442	2,887,271	1,806,148	1,701,228
	Miniasm/Racon	6,404,411	5,784,482	5,531,680	4,801,163	4,607,407	3,091,343	2,939,220	2,938,287	1,750,797	2,356,451
	Raven	6,431,899	5,692,207	5,427,572	4,798,621	4,572,192	4,202,171	2,937,267	2,872,322	1,746,441	1,838,559
	Redbean	6,481,326	6,019,522	5,397,115	6,241,523	4,697,676	5,367,318	3,055,771	2,727,082	1,304,726	2,007,331
	Shasta	6,451,477	5,591,706	5,403,949	4,856,338	4,696,934	4,245,065	2,952,549	2,881,942	1,604,863	1,666,266
	Reference	6,441,924	5,645,678	5,438,085	4,808,521	4,581,781	4,202,171	2,939,733	2,757,659	1,750,177	1,641,481
GC content (%)	Canu	66.33	35.53	50.59	52.35	56.64	28.05	38.09	32.90	31.70	30.93
	Flye	66.33	34.76	50.16	51.87	56.41	27.45	37.14	32.17	30.71	29.57
	Miniasm/Racon	66.37	35.40	50.66	52.36	56.75	22.65	37.97	32.83	31.36	30.24
	Raven	66.36	35.42	50.63	52.37	56.87	28.07	37.97	32.86	31.37	30.42
	Redbean	66.16	35.65	50.61	52.26	56.74	28.05	38.06	32.86	31.74	31.52
	Shasta	66.35	35.35	50.52	52.36	56.69	27.43	37.83	32.58	31.54	30.50
	Reference	66.29	35.50	50.50	52.21	56.73	28.07	37.98	32.84	31.41	30.55

^a^ cir., circular contigs; underlined cir. (cir.), the chromosomes were circularized. ^b^ N.A., not applicable.

**Table 4:** Numbers of virulence genes in bacterial strains with mediocre-quality reads compared to their corresponding reference genomes, as predicted based on their Oxford Nanopore long-read assemblies using different long-read assemblers.

Assembler	Numbers of Virulence Genes									
	*Pseudomonas aeruginosa* PAO1	*Escherichia coli* O157:H7 Sakai	*Bacillus anthracis* Ames Ancestor	*Klebsiella variicola* DSM 15968	*Salmonella* Typhimurium LT2	*Cronobacter sakazakii* ATCC 29544	*Clostridium botulinum* CDC_1632	*Listeria monocytogenes* EGD-e	*Staphylococcus aureus* TW20	*Campylobacter jejuni* NCTC 11168
Canu	188	125	13	10	118	2	0	32	68	119
Flye	188	126	13	10	118	2	0	32	68	118
Miniasm/Racon	188	119	13	10	110	2	0	32	67	118
Raven	188	119	13	10	110	2	0	32	68	118
Redbean	188	121	13	8	117	2	0	32	65	119
Shasta	188	122	13	9	108	1	0	32	68	118
Reference	188	126	13	10	118	2	0	32	68	118

**Table 5:** Numbers of virulence genes in bacterial strains with low-quality reads compared to their corresponding reference genomes, as predicted based on their Oxford Nanopore long-read assemblies using different long-read assemblers.

Assembler	Numbers of Virulence Genes									
	*Pseudomonas aeruginosa* PAO1	*Escherichia coli* O157:H7 Sakai	*Bacillus anthracis* Ames Ancestor	*Klebsiella variicola* DSM 15968	*Salmonella* Typhimurium LT2	*Cronobacter sakazakii* ATCC 29544	*Clostridium botulinum* CDC_1632	*Listeria monocytogenes* EGD-e	*Staphylococcus aureus* TW20	*Campylobacter jejuni* NCTC 11168
Canu	N.A.^a^	N.A.	N.A.	N.A.	N.A.	N.A.	N.A.	N.A.	N.A.	N.A.
Flye	1	N.A.	2	0	0	0	0	N.A.	0	N.A.
Miniasm/Racon	4	9	1	0	43	0	0	12	13	9
Raven	240	122	13	8	109	2	0	32	65	118
Redbean	1	0	0	0	0	0	0	0	0	N.A.
Shasta	N.A.	N.A.	N.A.	N.A.	N.A.	N.A.	N.A.	N.A.	N.A.	N.A.
Reference	188	126	13	10	118	2	0	32	68	118

^a^ N.A., not applicable.

**Table 6:** Numbers of virulence genes in bacterial strains with real reads compared to their corresponding reference genomes, as predicted based on their Oxford Nanopore long-read assemblies using different long-read assemblers.

Assembler	Numbers of Virulence Genes									
	*P seudomonas aeruginosa* CFSAN084950	*Bacillus paranthracis* CFSAN068816	*Escherichia coli* O157:H7 CFSAN076619	*Salmonella* Bareilly CFSAN000189	*Cronobacter sakazakii* CFSAN068773	*Clostridium botulinum* CFSAN034200	*Listeria monocytogenes* CFSAN023468	*Staphylococcus aureus* CFSAN007894	*Campylobacter coli* CFSAN032805	*Campylobacter jejuni* NCTC 11168
Canu	231	5	46	108	2	1	37	66	68	119
Flye	232	5	45	105	2	1	35	66	66	118
Miniasm/Racon	232	5	46	109	2	1	37	66	72	119
Raven	232	5	46	109	2	1	37	66	71	119
Redbean	232	23	46	108	2	1	35	56	55	102
Shasta	232	5	46	101	2	1	37	65	57	117
Reference	233	5	36	109	2	1	37	63	76	118

**Table 7:** Multilocus sequence typing (MLST) of bacterial strains with mediocre-quality reads compared to their corresponding reference genomes, as predicted based on their Oxford Nanopore long-read assemblies using different long-read assemblers.

Assembler	MLST									
	*Pseudomonas aeruginosa* PAO1	*Escherichia coli* O157:H7 Sakai	*Bacillus anthracis* Ames Ancestor	*Klebsiella variicola* DSM 15968	*Salmonella* Typhimurium LT2	*Cronobacter sakazakii* ATCC 29544	*Clostridium botulinum* CDC_1632	*Listeria monocytogenes* EGD-e	*Staphylococcus aureus* TW20	*Campylobacter jejuni* NCTC 11168
Canu	+ ^a^	+	+	+	+	+	−	+	+	+
Flye	+	+	−	+	−	−	−	−	−	−
Miniasm/Racon	+	+	+	+	+	+	+	+	+	+
Raven	+	+	+	+	+	+	−	+	+	+
Redbean	−	−	−	−	−	+	−	−	+	−
Shasta	−	−	−	−	−	−	−	−	−	−
Reference	+	+	+	+	+	+	+	+	+	+

^a^ +, accurately typed; −, not typed.

**Table 8:** Multilocus sequence typing (MLST) of bacterial strains with low-quality reads compared to their corresponding reference genomes, as predicted based on their Oxford Nanopore long-read assemblies using different long-read assemblers.

Assembler	MLST									
	*Pseudomonas aeruginosa* PAO1	*Escherichia coli* O157:H7 Sakai	*Bacillus anthracis* Ames Ancestor	*Klebsiella variicola* DSM 15968	*Salmonella* Typhimurium LT2	*Cronobacter sakazakii* ATCC 29544	*Clostridium botulinum* CDC_1632	*Listeria monocytogenes* EGD-e	*Staphylococcus aureus* TW20	*Campylobacter jejuni* NCTC 11168
Canu	N.A. ^b^	N.A.	N.A.	N.A.	N.A.	N.A.	N.A.	N.A.	N.A.	N.A.
Flye	− ^a^	N.A.	−	−	−	−	−	N.A.	−	N.A.
Miniasm/Racon	−	−	−	−	−	−	−	−	−	−
Raven	−	+	−	+	−	−	−	−	−	−
Redbean	−	−	−	−	−	−	−	−	−	N.A.
Shasta	N.A.	N.A.	N.A.	N.A.	N.A.	N.A.	N.A.	N.A.	N.A.	N.A.
Reference	+	+	+	+	+	+	+	+	+	+

^a^ +, accurately typed; −, not typed. ^b^ N.A., not applicable.

**Table 9:** Multilocus sequence typing (MLST) of bacterial strains with real reads compared to their corresponding reference genomes, as predicted based on their Oxford Nanopore long-read assemblies using different long-read assemblers.

Assembler	MLST									
	*P seudomonas aeruginosa* CFSAN084950	*Bacillus paranthracis* CFSAN068816	*Escherichia coli* O157:H7 CFSAN076619	*Salmonella* Bareilly CFSAN000189	*Cronobacter sakazakii* CFSAN068773	*Clostridium botulinum* CFSAN034200	*Listeria monocytogenes* CFSAN023468	*Staphylococcus aureus* CFSAN007894	*Campylobacter coli* CFSAN032805	*Campylobacter jejuni* NCTC 11168
Canu	+ ^a^	+	+	+	+	+	+	+	−	−
Flye	+	−	−	−	+	−	−	−	−	−
Miniasm/Racon	+	+	+	+	+	−	+	+	+	+
Raven	+	+	+	+	+	−	+	+	+	+
Redbean	+	+	+	+	+	+	+	+	−	−
Shasta	+	+	−	−	+	+	+	+	−	−
Reference	+	+	+	+	+	+	+	+	+	+

^a^ +, accurately typed; −, not typed.

**Table 10:** Bacterial strains with simulated Oxford Nanopore long reads.

Strain	GenBank Accession	RefSeq Category	Sequencing Technology	Assembly Method
*Pseudomonas aeruginosa* PAO1	GCA_000006765.1	Reference genome	N.A. ^a^	N.A.
*Escherichia coli* O157:H7 Sakai	GCA_000008865.2	Reference genome	Illumina MiSeq; PacBio RS II	N.A.
*Bacillus anthracis* Ames Ancestor	GCA_000008445.1	Representative genome	N.A.	N.A.
*Klebsiella variicola* DSM 15968	GCA_000828055.2	N.A.	PacBio	HGAP 2.1.1
*Salmonella* Typhimurium LT2	GCA_000006945.2	Reference genome	N.A.	N.A.
*Cronobacter sakazakii* ATCC 29544	GCA_000982825.1	N.A.	PacBio	HGAP 2.0
*Clostridium botulinum* CDC_1632	GCA_001889325.1	N.A.	Illumina; PacBio	Velvet 1.2.08; HGAP 3.0; Phrap SPS 4.24
*Listeria monocytogenes* EGD-e	GCA_000196035.1	Reference genome	N.A.	N.A.
*Staphylococcus aureus* TW20	GCA_000027045.1	N.A.	N.A.	N.A.
*Campylobacter jejuni* NCTC 11168	GCA_000009085.1	Reference genome	N.A.	N.A.

^a^ N.A., not applicable.

**Table 11:** Bacterial strains with real Oxford Nanopore long reads.

Strain	Reference Genome			Oxford Nanopore Sequencing	
	GenBank Accession	Sequencing Technology	Assembly Method	Run Accession	Platform
*Pseudomonas aeruginosa* CFSAN084950	GCA_009648875.1	Illumina MiSeq; Oxford Nanopore GridION	SPAdes 3.12.0; Canu 1.7	SRR10340796	GridION
*Bacillus paranthracis* CFSAN068816	GCA_009648955.1	Illumina MiSeq; Oxford Nanopore GridION	SPAdes 3.12.0; Canu 1.7	SRR10340799	GridION
*Escherichia coli* O157:H7 CFSAN076619	GCA_009650175.1	Illumina MiSeq; Oxford Nanopore GridION	SPAdes 3.12.0; Canu 1.7	SRR10346075	GridION
*Salmonella* Bareilly CFSAN000189	GCA_009648715.1	Illumina MiSeq; Oxford Nanopore GridION	SPAdes 3.12.0; Canu 1.7	SRR10337242	GridION
*Cronobacter sakazakii* CFSAN068773	GCA_009648895.1	Illumina MiSeq; Oxford Nanopore GridION	SPAdes 3.12.0; Canu 1.7	SRR10340800	GridION
*Clostridium botulinum* CFSAN034200	GCA_003345335.1	Illumina MiSeq; Oxford Nanopore MinION	SPAdes 3.11.1; Canu 1.7	SRR7530167	MinION
*Listeria monocytogenes* CFSAN023468	GCA_009648615.1	Illumina MiSeq; Oxford Nanopore GridION	SPAdes 3.12.0; Canu 1.7	SRR10336615	GridION
*Staphylococcus aureus* CFSAN007894	GCA_002633865.1	PacBio	HGAP v. 3.0	SRR10346110	GridION
*Campylobacter coli* CFSAN032805	GCA_009649055.1	Illumina MiSeq; Oxford Nanopore GridION	SPAdes 3.12.0; Canu 1.7	SRR10342326	GridION
*Campylobacter jejuni* NCTC 11168	GCA_000009085.1	N.A. ^a^	N.A.	ERR2722109	MinION

^a^ N.A., not applicable.

## Supplementary Materials

Supplementary Materials can be found at https://www.mdpi.com/1422-0067/21/23/9161/s1.

## Abbreviations

AMR	Antimicrobial resistance
ARGs	Antimicrobial resistance genes
AWS	Amazon Web Services
BUSCOs	Benchmarking universal single-copy orthologs
cgMLST	Core-genome multilocus sequence typing
FDA	U.S. Food and Drug Administration
MGEs	Mobile genetic elements
MLST	Multilocus sequence typing
NCBI	National Center for Biotechnology Information
OLC	Overlap–layout–consensus
RefSeq	Reference Sequence
SNPs	Single-nucleotide polymorphisms
SRA	Sequence Read Archive
wgMLST	Whole-genome multilocus sequence typing
WGS	Whole-genome sequencing
VFDB	Virulence Factors Database
