# Supplementary material for: Benchmarking Long-Read Assemblers for Genomic Analyses of Bacterial Pathogens Using Oxford Nanopore Sequencing
Source: Int J Mol Sci. 2020 Dec 1;21(23):9161. doi: 10.3390/ijms21239161 (PMC7730629; doi:10.3390/ijms21239161)
Supplement: Supplementary file 1 [file ijms-21-09161-s001.zip › ijms-976706/Supplementary Table S1.docx]

**Supplementary Table S1.** Complete benchmarking universal single-copy orthologs (BUSCOs) of Oxford Nanopore long-read assemblies of bacterial strains with mediocre-quality reads using different long-read assemblers

| Assembler | Complete BUSCOs (%) | | | | | | | | | | |
| --- | --- | --- | --- | --- | --- | --- | --- | --- | --- | --- | --- |
|  | ***Pseudomonas aeruginosa* PAO1** | ***Escherichia coli* O157:H7 Sakai** | ***Bacillus anthracis* Ames Ancestor** | ***Klebsiella variicola* DSM 15968** | ***Salmonella* Typhimurium LT2** | ***Cronobacter sakazakii* ATCC 29544** | ***Clostridium botulinum* CDC_1632** | ***Listeria monocytogenes* EGD-e** | ***Staphylococcus aureus* NCTC 8325** | ***Campylobacter jejuni* NCTC 11168** | **Average** |
| Canu | 51.4 | 43.2 | 23.6 | 43.2 | 44.6 | 46.6 | 8.1 | 25.7 | 22.3 | 9.5 | 31.8 |
| Flye | 54.7 | 20.3 | 6.1 | 29.8 | 25.7 | 23.0 | 2.0 | 4.7 | 4.1 | 0.7 | 17.1 |
| Miniasm/  Racon | 75.7 | 73.6 | 53.4 | 70.9 | 62.2 | 73.6 | 36.5 | 50.7 | 54.8 | 23.0 | 57.4 |
| Raven | 79.7 | 76.4 | 58.8 | 77.0 | 72.3 | 73.6 | 49.3 | 59.5 | 59.5 | 30.4 | 63.7 |
| Redbean | 14.9 | 18.9 | 6.8 | 16.9 | 13.5 | 10.8 | 3.4 | 4.1 | 7.4 | 6.8 | 10.4 |
| Shasta | 10.8 | 13.5 | 10.8 | 12.2 | 13.5 | 12.8 | 2.0 | 6.1 | 6.8 | 4.7 | 9.3 |
| Reference | 100.0 | 100.0 | 99.3 | 100.0 | 99.3 | 100.0 | 99.3 | 100.0 | 100.0 | 88.5 | 98.6 |

^a^N.A., not applicable.
