# Supplementary material for: Benchmarking Long-Read Assemblers for Genomic Analyses of Bacterial Pathogens Using Oxford Nanopore Sequencing
Source: Int J Mol Sci. 2020 Dec 1;21(23):9161. doi: 10.3390/ijms21239161 (PMC7730629; doi:10.3390/ijms21239161)
Supplement: Supplementary file 1 [file ijms-21-09161-s001.zip › ijms-976706/Supplementary Table S10.docx]

**Supplementary Table S10.** Antimicrobial resistance (AMR) genotypes and phenotypes of bacterial strains with mediocre-quality reads compared to their corresponding reference genomes, as predicted based on their Oxford Nanopore long-read assemblies using different long-read assemblers^a^

| Assembler | AMR | | | | | | | | | | | |
| --- | --- | --- | --- | --- | --- | --- | --- | --- | --- | --- | --- | --- |
|  | ***Pseudomonas aeruginosa* PAO1** | | ***Bacillus anthracis* Ames Ancestor** | | ***Klebsiella variicola* DSM 15968** | | ***Listeria monocytogenes* EGD-e** | | ***Staphylococcus aureus* TW20** | | ***Campylobacter jejuni* NCTC 11168** | |
|  | **Genotype** | **Phenotype** | **Genotype** | **Phenotype** | **Genotype** | **Phenotype** | **Genotype** | **Phenotype** | **Genotype** | **Phenotype** | **Genotype** | **Phenotype** |
| Canu | *aph(3')-IIb*  *blaOXA-50*  *blaPAO*  *catB7*  *fosA* | Kanamycin  Ampicillin  Amoxicillin/Clavulanic acid  Cefoxitinceftriaxone  Chloramphenicol  Fosfomycin | *fosB2* | Fosfomycin | *blaLEN17* | Ampicillin | *fosX* | Fosfomycin | *aac(6')-aph(2'')*  *ant(6)-Ia*  *aph(3')-III*  *blaZ*  *dfrG*  *erm(33)*  *erm(A)*  *mecA*  *spc*  *tet(K)*  *tet(M)* | Gentamicin  Streptomycin  Kanamycin  Ampicillin  Trimethoprim  Erythromycin  Azithromycin  Spectinomycin  Tetracycline | *blaOXA-61* | Ampicillin |
| Flye | *aph(3')-IIb*  *blaOXA-50*  *blaPAO*  *catB7*  *fosA* | Kanamycin  Ampicillin  Amoxicillin/Clavulanic acid  Cefoxitin  Ceftriaxone  Chloramphenicol  Fosfomycin | -^b^ | - | *blaLEN17* | Ampicillin | *fosX* | Fosfomycin | *aac(6')-aph(2'')*  *ant(6)-Ia*  *aph(3')-III*  *dfrG*  *erm(A)*  *mecA*  *spc*  *tet(K)*  *tet(M)* | Gentamicin  Streptomycin  Kanamycin  Trimethoprim  Erythromycin  Azithromycin  Spectinomycin  Tetracycline | - | - |
| Miniasm/Racon | *aph(3')-IIb*  *blaOXA-50*  *blaPAO*  *catB7*  *fosA* | Kanamycin  Ampicillin, Amoxicillin/Clavulanic acid  Cefoxitin  Ceftriaxone  Chloramphenicol  Fosfomycin | *fosB2* | Fosfomycin | *blaLEN17* | Ampicillin | *fosX* | Fosfomycin | *aac(6')-aph(2'')*  *ant(6)-Ia*  *aph(3')-III*  *blaZ*  *dfrG*  *erm(A)*  *mecA*  *spc*  *tet(K)*  *tet(M)* | Gentamicin  Streptomycin  Kanamycin  Ampicillin  Trimethoprim  Erythromycin  Azithromycin  Spectinomycin  Tetracycline | *blaOXA-61* | Ampicillin |
| Raven | *aph(3')-IIb*  *blaOXA-50*  *blaPAO*  *catB7*  *fosA* | Kanamycin  Ampicillin  Amoxicillin/Clavulanic acid  Cefoxitin  Ceftriaxone  Chloramphenicol  Fosfomycin | *fosB2* | Fosfomycin | *blaLEN17* | Ampicillin | *fosX* | Fosfomycin | *aac(6')-aph(2'')*  *ant(6)-Ia*  *aph(3')-III*  *blaZ*  *dfrG*  *erm(A)*  *mecA*  *spc*  *tet(K)*  *tet(M)* | Gentamicin  Streptomycin  Kanamycin  Ampicillin  Trimethoprim  Erythromycin  Azithromycin  Spectinomycin  Tetracycline | *blaOXA-61* | Ampicillin |
| Redbean | *catB7* | Chloramphenicol | *fosB2* | Fosfomycin | - | - | - | - | *blaZ* | Ampicillin | *blaOXA-61* | Ampicillin |
| Shasta | *aph(3')-IIb*  *blaPAO* | Kanamycin  Ampicillin  Amoxicillin/Clavulanic acid  Cefoxitin  Ceftriaxone | *fosB2* | Fosfomycin | - | - | *fosX* | Fosfomycin | *aph(3')-III*  *blaZ*  *dfrG*  *erm(33)*  *spc*  *tet(M)* | Kanamycin  Ampicillin  Trimethoprim  Erythromycin  Azithromycin  Spectinomycin  Tetracycline | *blaOXA-61* | Ampicillin |
| Reference | *aph(3')-IIb*  *blaOXA-50*  *blaPAO*  *catB7*  *fosA* | Kanamycin  Ampicillin  Amoxicillin/Clavulanic acid  Cefoxitin  Ceftriaxone  Chloramphenicol  Fosfomycin | *fosB2* | Fosfomycin | *blaLEN17* | Ampicillin | *fosX* | Fosfomycin | *aac(6')-aph(2'')*  *ant(6)-Ia*  *aph(3')-III*  *blaZ*  *dfrG*  *erm(A)*  *mecA*  *spc*  *tet(K)*  *tet(M)* | Gentamicin  Streptomycin  Kanamycin  Ampicillin  Trimethoprim  Erythromycin  Azithromycin  Spectinomycin  Tetracycline | *blaOXA-61* | Ampicillin |

^a^ARGs or chromosomal point mutations were not identified in any Oxford Nanopore assemblies of *Escherichia coli* O157:H7 Sakai, *Salmonella* Typhimurium LT2, *Cronobacter sakazakii* ATCC 29544, and *Clostridium botulinum* CDC_1632.

^b^-, not detected.
