# Supplementary material for: Benchmarking Long-Read Assemblers for Genomic Analyses of Bacterial Pathogens Using Oxford Nanopore Sequencing
Source: Int J Mol Sci. 2020 Dec 1;21(23):9161. doi: 10.3390/ijms21239161 (PMC7730629; doi:10.3390/ijms21239161)
Supplement: Supplementary file 1 [file ijms-21-09161-s001.zip › ijms-976706/Supplementary Table S12.docx]

**Supplementary Table S12.** Antimicrobial resistance (AMR) genotypes and phenotypes of bacterial strains with real reads compared to their corresponding reference genomes, as predicted based on their Oxford Nanopore long-read assemblies using different long-read assemblers^a^

| Assembler | AMR | | | | | | | | | |
| --- | --- | --- | --- | --- | --- | --- | --- | --- | --- | --- |
|  | ***Pseudomonas aeruginosa* CFSAN084950** | | ***E. coli* O157:H7 CFSAN076619** | | ***Staphylococcus aureus* CFSAN007894** | | ***Campylobacter coli* CFSAN032805** | | ***Campylobacter jejuni* NCTC 11168** | |
|  | **Genotype** | **Phenotype** | **Genotype** | **Phenotype** | **Genotype** | **Phenotype** | **Genotype** | **Phenotype** | **Genotype** | **Phenotype** |
| Canu | *aph(3')-IIb*  *blaOXA-50*  *blaPAO*  *catB7*  *fosA* | Kanamycin  Ampicillin  Amoxicillin/Clavulanic acid  Cefoxitin  Ceftriaxone  Chloramphenicol  Fosfomycin | *aph(3')-III* | Kanamycin | *mph(C)*  *msr(A)*  *msr(A)*  *tet(K)* | Erythromycin  Azithromycin  Tetracycline | *aph(2'')-Ig*  *aph(3')-III*  *tet(O)* | Kanamycin  Tetracycline | -^b^ | - |
| Flye | *aph(3')-IIb*  *blaOXA-50*  *blaPAO*  *catB7*  *fosA* | Kanamycin  Ampicillin  Amoxicillin/Clavulanic acid  Cefoxitin  Ceftriaxone  Chloramphenicol  Fosfomycin | - | - | *mph(C)* | Erythromycin | *aph(3')-III* | Kanamycin | - | - |
| Miniasm/Racon | *aph(3')-IIb*  *blaOXA-50*  *blaPAO*  *catB7*  *fosA* | Kanamycin  Ampicillin  Amoxicillin/Clavulanic acid  Cefoxitin  Ceftriaxone  Chloramphenicol  Fosfomycin | *aph(3')-III* | Kanamycin | *mph(C), msr(A), tet(K)* | Erythromycin  Azithromycin  Tetracycline | *aph(2'')-Ig*  *aph(3')-III*  *tet(O)* | Kanamycin  Tetracycline | *blaOXA-61* | Ampicillin |
| Raven | *aph(3')-IIb*  *blaOXA-50*  *blaPAO*  *catB7*  *fosA* | Kanamycin  Ampicillin  Amoxicillin/Clavulanic acid  Cefoxitin  Ceftriaxone  Chloramphenicol  Fosfomycin | *aph(3')-III* | Kanamycin | *mph(C), msr(A), tet(K)* | Erythromycin  Azithromycin  Tetracycline | *aph(2'')-Ig*  *aph(3')-IIIa*  *tet(O)* | Kanamycin  Tetracycline | *blaOXA-61* | Ampicillin |
| Redbean | *aph(3')-IIb*  *blaOXA-50*  *blaPAO*  *catB7*  *fosA* | Kanamycin  Ampicillin  Amoxicillin/Clavulanic acid  Cefoxitin  Ceftriaxone  Chloramphenicol  Fosfomycin | *aph(3')-III* | Kanamycin | *tet(K)* | Tetracycline | *aph(2'')-Ig*  *aph(3')-III*  *tet(O)* | Kanamycin  Tetracycline | - | - |
| Shasta | *aph(3')-IIb*  *blaOXA-50*  *blaPAO*  *catB7*  *fosA* | Kanamycin  Ampicillin  Amoxicillin/Clavulanic acid  Cefoxitin  Ceftriaxone  Chloramphenicol  Fosfomycin | *-* | *-* | *mph(C)*  *msr(A)* | Erythromycin  Azithromycin | *aph(2'')-Ig*  *aph(3')-III*  *tet(O)* | Kanamycin  Tetracycline | - | - |
| Reference | *aph(3')-IIb*  *blaOXA-50*  *blaPAO*  *catB7*  *fosA* | Kanamycin  Ampicillin  Amoxicillin/Clavulanic acid  Cefoxitin  Ceftriaxone  Chloramphenicol  Fosfomycin | *aph(3')-III* | Kanamycin | *blaZ* | Ampicillin | *aph(2'')-Ig*  *aph(3')-III*  *blaOXA-61*  *tet(O)* | Kanamycin  Ampicillin  Tetracycline | *blaOXA-61* | Ampicillin |

^a^ARGs or chromosomal point mutations were not identified in any Oxford Nanopore assemblies of *S.* Bareilly CFSAN000189, *C. sakazakii* CFSAN068773, *C. botulinum* CFSAN034200, or *L. monocytogenes* CFSAN023468.

^b^-, not detected.
