# Supplementary material for: Benchmarking Long-Read Assemblers for Genomic Analyses of Bacterial Pathogens Using Oxford Nanopore Sequencing
Source: Int J Mol Sci. 2020 Dec 1;21(23):9161. doi: 10.3390/ijms21239161 (PMC7730629; doi:10.3390/ijms21239161)
Supplement: Supplementary file 1 [file ijms-21-09161-s001.zip › ijms-976706/Supplementary Table S13.docx]

**Supplementary Table S13.** Thirty closely related *Listeria monocytogenes* strains of *L. monocytogenes* EGD-e selected based on the single nucleotide polymorphisms (SNP) (Number of SNPs<500) and core-genome multilocus sequence typing (cgMLST) (Different alleles<500) strategies

| Strain | GenBank or run accession |
| --- | --- |
| ICDC-LM1871 | GCA_009788285.1 |
| ICDC-LM1872 | GCA_009788295.1 |
| ICDC-LM1873 | GCA_009788365.1 |
| 5KSM | GCA_000978685.1 |
| 10 | GCA_008807475.1 |
| 2008-911 | GCA_001866445.1 |
| 33761 | SRR7629005^a^ |
| A538 | GCA_001711785.1 |
| AL4E | GCA_002557815.1 |
| AUSMDU00000224 | GCA_009664775.1 |
| CDPHFDLB-F15M03674-2a | GCA_002488625.1 |
| CFSAN035177 | GCA_003002335.1 |
| CFSAN035186 | GCA_003002415.1 |
| CFSAN035190 | GCA_003004055.1 |
| CFSAN044778 | GCA_002524825.1 |
| CFSAN044803 | GCA_002524305.1 |
| FRR B 2941 | GCA_002114845.1 |
| FSL R8-6134 | GCA_000727385.1 |
| Lm233 | GCA_001709465.1 |
| MF4624 | GCA_002843525.1 |
| MF4626 | GCA_002831485.1 |
| N11-1837 | GCA_003589425.1 |
| NRRL B-33344 | GCA_003608875.1 |
| p959-10 | GCA_003011635.1 |
| PNUSAL000190 | GCA_003606775.1 |
| SCPM-O-B-8698 | GCA_011881945.1 |
| SHL12-2 | GCA_002000265.1 |
| SHL014 | GCA_002760575.1 |
| V1053 | GCA_002879335.1 |
| W1041 | GCA_002878715.1 |

^a^Illumina short reads were assembled using SPAdes 3.14.1.
