# Supplementary material for: Benchmarking Long-Read Assemblers for Genomic Analyses of Bacterial Pathogens Using Oxford Nanopore Sequencing
Source: Int J Mol Sci. 2020 Dec 1;21(23):9161. doi: 10.3390/ijms21239161 (PMC7730629; doi:10.3390/ijms21239161)
Supplement: Supplementary file 1 [file ijms-21-09161-s001.zip › ijms-976706/Supplementary Table S14.docx]

**Supplementary Table S14.** Thirty distantly related *Listeria monocytogenes* strains of *L. monocytogenes* EGD-e selected based on the single nucleotide polymorphisms (SNP) strategy (Number of SNPs>500)

| Strain | GenBank or run accession |
| --- | --- |
| 06B00637F-1 | GCA_004468665.1 |
| 08-6056 | GCA_002213585.1 |
| 944 | GCA_001463975.1 |
| 2012-0070 | GCA_001866455.1 |
| 2981 | GCA_001658325.1 |
| 52869 | GCA_008807895.1 |
| 673671 | GCA_004476145.1 |
| BCW_4786 | GCA_001828155.1 |
| BS-26 | GCA_001880165.1 |
| CFSAN010068 | GCA_001548635.1 |
| CFSAN028749 | GCA_004493035.1 |
| CFSAN045927 | GCA_002526965.1 |
| FDA00006665 | SRR2585477^a^ |
| FDA00014772 | [SRR10298063](https://trace.ncbi.nlm.nih.gov/Traces/sra/?run=SRR10298063) |
| FDA00015126 | SRR11032501 |
| FNW19G43 | GCA_003590245.1 |
| FSLF2-661 | GCA_003679675.1 |
| G6054 | GCA_003417835.1 |
| LM-F-73 | GCA_003189325.1 |
| LmNG2 | GCA_900174555.1 |
| M2294 | GCA_002879435.1 |
| Marseille-Q1049 | GCA_902506745.1 |
| MOD1_LS884 | GCA_000787895.1 |
| N12-0728 | GCA_003588675.1 |
| N12-0935 | GCA_004768685.1 |
| N16-0855 | GCA_003097435.1 |
| NRRL_B-57056 | GCA_001749905.1 |
| p961-18 | GCA_003011755.1 |
| PNUSAL000391 | GCA_003606515.1 |
| PNUSAL006065 | GCA_009233525.1 |

^a^Illumina short reads were assembled using SPAdes 3.14.1.
