# Supplementary material for: Benchmarking Long-Read Assemblers for Genomic Analyses of Bacterial Pathogens Using Oxford Nanopore Sequencing
Source: Int J Mol Sci. 2020 Dec 1;21(23):9161. doi: 10.3390/ijms21239161 (PMC7730629; doi:10.3390/ijms21239161)
Supplement: Supplementary file 1 [file ijms-21-09161-s001.zip › ijms-976706/Supplementary Table S15.docx]

**Supplementary Table S15.** Thirty closely related *Salmonella* Bareilly strains of *S.* Bareilly CFSAN000189 selected based on the single nucleotide polymorphisms (SNP) strategy (Number of SNPs<500)

| Strain | GenBank or run accession |
| --- | --- |
| 191871 | GCA_003912215.1 |
| 218465 | GCA_004185915.1 |
| 266962 | GCA_003908315.1 |
| 267815 | GCA_003912285.1 |
| 282611 | GCA_003888935.1 |
| 491026 | GCA_003888695.1 |
| 509960 | GCA_003890615.1 |
| 611465 | GCA_003922945.1 |
| BCW_2645 | GCA_002066135.1 |
| CFSAN000186 | GCA_000749435.1 |
| CFSAN000191 | GCA_003627015.2 |
| CFSAN000211 | GCA_000698715.2 |
| CFSAN000232 | GCA_000748625.1 |
| CFSAN000661 | GCA_009648835.1 |
| CFSAN000662 | GCA_000748985.1 |
| CFSAN000955 | GCA_000748425.1 |
| CFSAN000959 | GCA_000748485.1 |
| CFSAN000961 | GCA_004847745.1 |
| CFSAN000964 | GCA_000749315.1 |
| CFSAN000969 | GCA_000749395.1 |
| CFSAN000970 | GCA_004847545.1 |
| CFSAN001090 | GCA_000758005.1 |
| CFSAN001102 | GCA_000748725.1 |
| CFSAN001108 | GCA_000748765.1 |
| CFSAN001109 | GCA_000748785.1 |
| CFSAN001118 | GCA_004847145.1 |
| CFSAN001140 | GCA_004847005.1 |
| FAR0125 | GCA_003869735.1 |
| FDA00009340 | SRR2534086^a^ |
| PNUSAS048129 | GCA_003895875.1 |

^a^Illumina short reads were assembled using SPAdes 3.14.1.
