# Supplementary material for: Benchmarking Long-Read Assemblers for Genomic Analyses of Bacterial Pathogens Using Oxford Nanopore Sequencing
Source: Int J Mol Sci. 2020 Dec 1;21(23):9161. doi: 10.3390/ijms21239161 (PMC7730629; doi:10.3390/ijms21239161)
Supplement: Supplementary file 1 [file ijms-21-09161-s001.zip › ijms-976706/Supplementary Table S16.docx]

**Supplementary Table S16.** Eleven closely related *Campylobacter jejuni* strains of *C. jejuni* NCTC 11168 selected based on the single nucleotide polymorphisms (SNP) strategy (Number of SNPs<500)

| Strain | GenBank accession |
| --- | --- |
| 11168H/araE | GCA_002238375.1 |
| 11168H/lacY | GCA_002224325.1 |
| ERS1434026 | GCA_000254315.2 |
| FDAARGOS_263 | GCA_002209025.1 |
| g113 | GCA_000633065.1 |
| NCTC 11168-BN148 | GCA_000304375.1 |
| NCTC 11168-K12E5 | GCA_000830775.1 |
| NCTC 11168-Kf1 | GCA_000830805.1 |
| NCTC 11168-mcK12E5 | GCA_000830825.1 |
| NCTC 11168-mfK12E5 | GCA_000830845.1 |
| NCTC 11168-rNRC | GCA_000830865.1 |
