# Supplementary material for: Benchmarking Long-Read Assemblers for Genomic Analyses of Bacterial Pathogens Using Oxford Nanopore Sequencing
Source: Int J Mol Sci. 2020 Dec 1;21(23):9161. doi: 10.3390/ijms21239161 (PMC7730629; doi:10.3390/ijms21239161)
Supplement: Supplementary file 1 [file ijms-21-09161-s001.zip › ijms-976706/Supplementary Table S17.docx]

**Supplementary Table S17.** Twenty distantly related *Campylobacter jejuni* strains of *C. jejuni* NCTC 11168 selected based on the single nucleotide polymorphisms (SNP) strategy (Number of SNPs>500) and twenty *Campylobacter* strains of other species

| Species | Strain | GenBank accession |
| --- | --- | --- |
| *Campylobacter jejuni* | CFSAN065302 | GCA_002806445.1 |
|  | CFSAN065320 | GCA_002805465.1 |
|  | CFSAN065369 | GCA_002804945.1 |
|  | CFSAN065379 | GCA_002805345.1 |
|  | CFSAN065387 | GCA_002805065.1 |
|  | CFSAN065392 | GCA_002804955.1 |
|  | CFSAN065405 | GCA_002804445.1 |
|  | CFSAN070903 | GCA_005303405.1 |
|  | CFSAN096308 | GCA_010362145.2 |
|  | CFSAN096313 | GCA_010359285.1 |
|  | CFSAN096317 | GCA_010225635.2 |
|  | CFSAN096319 | GCA_010359885.2 |
|  | CFSAN096328 | GCA_010225655.2 |
|  | CFSAN096330 | GCA_010358605.2 |
|  | CFSAN096334 | GCA_010012445.2 |
|  | CFSAN096335 | GCA_010359745.2 |
|  | CFSAN096345 | GCA_010359425.2 |
|  | I2019004398 | GCA_005211465.1 |
|  | I2019005837 | GCA_007740315.1 |
|  | PNUSAC014798 | GCA_010360725.1 |
| *Campylobacter gracilis* | ATCC 33236 | GCA_001190745.1 |
| *Campylobacter cuniculorum* | LMG 24588 | GCA_002104335.1 |
| *Campylobacter fetus* | NCTC 10354 | GCA_008271385.1 |
| *Campylobacter subantarcticus* | LMG 24377 | GCA_000816305.1 |
| *Campylobacter concisus* | ATCC 33237 | GCA_001298465.1 |
| *Campylobacter pinnipediorum* | RM17260 | GCA_002021925.1 |
| *Campylobacter helveticus* | ATCC 51209 | GCA_002080395.1 |
| *Campylobacter sputorum* | LMG 7795 | GCA_008245005.1 |
| *Campylobacter avium* | LMG 24591 | GCA_002238335.1 |
| *Campylobacter peloridis* | LMG 23910 | GCA_000816785.1 |
| *Campylobacter hominis* | ATCC BAA-381 | GCA_000017585.1 |
| *Campylobacter iguaniorum* | 1485E | GCA_000736415.1 |
| *Campylobacter lanienae* | NCTC 13004 | GCA_002139935.1 |
| *Campylobacter volucris* | LMG 24380 | GCA_008245045.1 |
| *Campylobacter hepaticus* | HV10 | GCA_001687475.2 |
| *Campylobacter insulaenigrae* | NCTC 12927 | GCA_000816185.1 |
| *Campylobacter curvus* | 525.92 | GCA_000017465.2 |
| *Campylobacter hyointestinalis* | LMG 9260 | GCA_001643955.1 |
| *Campylobacter armoricus* | CA639 | GCA_009036385.1 |
| *Campylobacter ureolyticus* | RIGS 9880 | GCA_001190755.1 |
