# Supplementary material for: Benchmarking Long-Read Assemblers for Genomic Analyses of Bacterial Pathogens Using Oxford Nanopore Sequencing
Source: Int J Mol Sci. 2020 Dec 1;21(23):9161. doi: 10.3390/ijms21239161 (PMC7730629; doi:10.3390/ijms21239161)
Supplement: Supplementary file 1 [file ijms-21-09161-s001.zip › ijms-976706/Supplementary Table S18.docx]

**Supplementary Table S18.** Thirty distantly related *Pseudomonas aeruginosa* strains of *P. aeruginosa* PAO1 selected based on the single nucleotide polymorphisms (SNP) strategy (Number of SNPs>500)

| Strain | GenBank accession |
| --- | --- |
| 519119 | GCA_007559105.1 |
| AES1R | GCA_004355145.1 |
| AG1 | GCA_009662315.1 |
| AR_0110 | GCA_003204335.1 |
| AR_0230 | GCA_002968695.1 |
| AR439 | GCA_003073895.1 |
| C7-25 | GCA_902703215.1 |
| DK2 | GCA_000271365.1 |
| F30658 | GCA_001516265.1 |
| FDAARGOS_570 | GCA_003813025.1 |
| H27930 | GCA_001516325.2 |
| JB2 | GCA_003060845.1 |
| N15-01092 | GCA_003571505.1 |
| NCGM257 | GCA_001547955.1 |
| Ocean-1155 | GCA_002237405.1 |
| PA_D2 | GCA_001721765.1 |
| PA7 | GCA_000017205.1 |
| PA83 | GCA_002215345.1 |
| PA99 | GCA_009498355.1 |
| Pa127 | GCA_002205355.1 |
| PABL048 | GCA_003411785.2 |
| PB350 | GCA_002812905.2 |
| PB368 | GCA_002812845.1 |
| SCVJan | GCA_001900225.1 |
| T2436 | GCA_009720405.1 |
| T52373 | GCA_001516005.1 |
| UCBPP-PA14 | GCA_000014625.1 |
| VIT PC9 | GCA_010694505.1 |
| Y71 | GCA_003408495.1 |
| YL84 | GCA_000524595.1 |
