# Supplementary material for: Benchmarking Long-Read Assemblers for Genomic Analyses of Bacterial Pathogens Using Oxford Nanopore Sequencing
Source: Int J Mol Sci. 2020 Dec 1;21(23):9161. doi: 10.3390/ijms21239161 (PMC7730629; doi:10.3390/ijms21239161)
Supplement: Supplementary file 1 [file ijms-21-09161-s001.zip › ijms-976706/Supplementary Table S19.docx]

**Supplementary Table S19.** Six closely related *Escherichia coli* O157:H7 strains of *E. coli* O157:H7 CFSAN076619 selected based on the whole-genome multilocus sequence typing (wgMLST) strategy (Different alleles<500)

| Strain | GenBank accession |
| --- | --- |
| 88.1042 | GCA_000316345.2 |
| 90.2281 | GCA_000316425.2 |
| 96.0428 | GCA_000316905.2 |
| EDL933 | GCA_000732965.1 |
| FDA504 | GCA_000303755.2 |
| FDAARGOS_292 | GCA_002206405.2 |
