# Supplementary material for: Benchmarking Long-Read Assemblers for Genomic Analyses of Bacterial Pathogens Using Oxford Nanopore Sequencing
Source: Int J Mol Sci. 2020 Dec 1;21(23):9161. doi: 10.3390/ijms21239161 (PMC7730629; doi:10.3390/ijms21239161)
Supplement: Supplementary file 1 [file ijms-21-09161-s001.zip › ijms-976706/Supplementary Table S2.docx]

**Supplementary Table S2.** Numbers of single nucleotide polymorphisms (SNPs) and indels in Oxford Nanopore long-read assemblies of bacterial strains with mediocre-quality reads using different long-read assemblers, as determined by aligning to their corresponding reference genomes, and expressed as SNPs and indels per one million bp of the reference genomes, respectively

| Assembler | *Pseudomonas aeruginosa* PAO1 | | *Escherichia coli* O157:H7 Sakai | | *Bacillus anthracis* Ames Ancestor | | *Klebsiella variicola* DSM 15968 | | *Salmonella* Typhimurium LT2 | | *Cronobacter sakazakii* ATCC 29544 | | *Clostridium botulinum* CDC_1632 | | *Listeria monocytogenes* EGD-e | | *Staphylococcus aureus* TW20 | | *Campylobacter jejuni* NCTC 11168 | | Average | |
| --- | --- | --- | --- | --- | --- | --- | --- | --- | --- | --- | --- | --- | --- | --- | --- | --- | --- | --- | --- | --- | --- | --- |
|  | **SNPs** | **Indels** | **SNPs** | **Indels** | **SNPs** | **Indels** | **SNPs** | **Indels** | **SNPs** | **Indels** | **SNPs** | **Indels** | **SNPs** | **Indels** | **SNPs** | **Indels** | **SNPs** | **Indels** | **SNPs** | **Indels** | **SNPs** | **Indels** |
| Canu | 13 | 2,110 | 9 | 3,450 | 5 | 5,224 | 8 | 3,222 | 11 | 3,473 | 8 | 2,860 | 3 | 6,637 | 5 | 5,036 | 11 | 4,350 | 2 | 7,981 | 8 | 4,434 |
| Flye | 136 | 1,803 | 108 | 5,734 | 75 | 10,424 | 125 | 4,301 | 126 | 5,665 | 95 | 4,631 | 150 | 14,170 | 58 | 11,082 | 66 | 10,446 | 124 | 19,197 | 106 | 8,745 |
| Miniasm/  Racon | 12 | 717 | 13 | 1,277 | 18 | 1,896 | 15 | 1,254 | 16 | 1,300 | 10 | 1,338 | 22 | 2,503 | 15 | 1,873 | 17 | 1,689 | 20 | 2,739 | 16 | 1,659 |
| Raven | 6 | 596 | 4 | 1,083 | 9 | 1,660 | 7 | 1,057 | 9 | 1,116 | 5 | 906 | 13 | 2,149 | 6 | 1,644 | 10 | 1,476 | 13 | 2,467 | 8 | 1,415 |
| Redbean | 1,900 | 17,230 | 1,564 | 15,467 | 1,688 | 21,810 | 1,942 | 17,016 | 1,758 | 22,428 | 1,713 | 15,870 | 1,542 | 19,810 | 1,751 | 22,713 | 1,909 | 9,890 | 1,240 | 20,556 | 1,701 | 18,279 |
| Shasta | 608 | 11,600 | 459 | 10,906 | 377 | 10,269 | 533 | 11,213 | 501 | 10,700 | 480 | 10,758 | 417 | 12,691 | 387 | 10,082 | 452 | 11,972 | 301 | 10,524 | 452 | 11,072 |
