# Supplementary material for: Benchmarking Long-Read Assemblers for Genomic Analyses of Bacterial Pathogens Using Oxford Nanopore Sequencing
Source: Int J Mol Sci. 2020 Dec 1;21(23):9161. doi: 10.3390/ijms21239161 (PMC7730629; doi:10.3390/ijms21239161)
Supplement: Supplementary file 1 [file ijms-21-09161-s001.zip › ijms-976706/Supplementary Table S3.docx]

**Supplementary Table S3.** Complete benchmarking universal single-copy orthologs (BUSCOs) of Oxford Nanopore long-read assemblies of bacterial strains with low-quality reads using different long-read assemblers

| Assembler | Complete BUSCOs (%) | | | | | | | | | | |
| --- | --- | --- | --- | --- | --- | --- | --- | --- | --- | --- | --- |
|  | ***Pseudomonas aeruginosa* PAO1** | ***Escherichia coli* O157:H7 Sakai** | ***Bacillus anthracis* Ames Ancestor** | ***Klebsiella variicola* DSM 15968** | ***Salmonella* Typhimurium LT2** | ***Cronobacter sakazakii* ATCC 29544** | ***Clostridium botulinum* CDC_1632** | ***Listeria monocytogenes* EGD-e** | ***Staphylococcus aureus* TW20** | ***Campylobacter jejuni* NCTC 11168** | **Average** |
| Canu | N.A. | N.A. | N.A. | N.A. | N.A. | N.A. | N.A. | N.A. | N.A. | N.A. | N.A. |
| Flye | 0.0 | N.A. | 0.0 | 0.0 | 0.0 | 0.0 | 0.0 | N.A. | 0.0 | N.A. | 0.0 |
| Miniasm/  Racon | 0.0 | 2.7 | 0.0 | 0.0 | 4.7 | 0.0 | 0.0 | 2.0 | 2.0 | 0.0 | 1.1 |
| Raven | 7.4 | 8.1 | 6.8 | 10.1 | 15.6 | 10.8 | 0.0 | 4.1 | 5.4 | 1.4 | 7.0 |
| Redbean | 0.0 | 0.0 | 0.0 | 0.0 | 0.0 | 0.0 | 0.0 | 0.0 | 0.0 | N.A. | 0.0 |
| Shasta | N.A. | N.A. | N.A. | N.A. | N.A. | N.A. | N.A. | N.A. | N.A. | N.A. | N.A. |
| Reference | 100.0 | 100.0 | 99.3 | 100.0 | 99.3 | 100.0 | 99.3 | 100.0 | 100.0 | 88.5 | 98.6 |

^a^N.A., not applicable.
