# Supplementary material for: Benchmarking Long-Read Assemblers for Genomic Analyses of Bacterial Pathogens Using Oxford Nanopore Sequencing
Source: Int J Mol Sci. 2020 Dec 1;21(23):9161. doi: 10.3390/ijms21239161 (PMC7730629; doi:10.3390/ijms21239161)
Supplement: Supplementary file 1 [file ijms-21-09161-s001.zip › ijms-976706/Supplementary Table S4.docx]

**Supplementary Table S4.** Numbers of single nucleotide polymorphisms (SNPs) and indels in Oxford Nanopore long-read assemblies of bacterial strains with low-quality reads using different long-read assemblers, as determined by aligning to their corresponding reference genomes, and expressed as SNPs and indels per one million bp of the reference genomes, respectively

| Assembler | *Pseudomonas aeruginosa* PAO1 | | *Escherichia coli* O157:H7 Sakai | | *Bacillus anthracis* Ames Ancestor | | *Klebsiella variicola* DSM 15968 | | *Salmonella* Typhimurium LT2 | | *Cronobacter sakazakii* ATCC 29544 | | *Clostridium botulinum* CDC_1632 | | *Listeria monocytogenes* EGD-e | | *Staphylococcus aureus* TW20 | | *Campylobacter jejuni* NCTC 11168 | | Average | |
| --- | --- | --- | --- | --- | --- | --- | --- | --- | --- | --- | --- | --- | --- | --- | --- | --- | --- | --- | --- | --- | --- | --- |
|  | **SNPs** | **Indels** | **SNPs** | **Indels** | **SNPs** | **Indels** | **SNPs** | **Indels** | **SNPs** | **Indels** | **SNPs** | **Indels** | **SNPs** | **Indels** | **SNPs** | **Indels** | **SNPs** | **Indels** | **SNPs** | **Indels** | **SNPs** | **Indels** |
| Canu | N.A.^a^ | N.A. | N.A. | N.A. | N.A. | N.A. | N.A. | N.A. | N.A. | N.A. | N.A. | N.A. | N.A. | N.A. | N.A. | N.A. | N.A. | N.A. | N.A. | N.A. | N.A. | N.A. |
| Flye | 1 | 15,089 | N.A. | N.A. | 1 | 21,339 | -^b^ | 14,114 | - | 12,820 | - | 9,611 | - | 21,257 | N.A. | N.A. | 3 | 0 | N.A. | N.A. | 2 | 13,461 |
| Miniasm/  Racon | 0 | 12,507 | 51 | 10,296 | 0 | 15,975 | 42 | 13,754 | 61 | 9,926 | 40 | 12,489 | 101 | 16,632 | 86 | 13.579 | 4 | 13,666 | 161 | 16,814 | 55 | 12,207 |
| Raven | 0 | 12,826 | 37 | 11,973 | 0 | 14,504 | 16 | 13,378 | 49 | 12,325 | 20 | 11,970 | 54 | 17,530 | 68 | 14.088 | 2 | 13,130 | 64 | 17,905 | 31 | 12,556 |
| Redbean | - | 977 | - | 3,428 | - | 0 | - | 0 | - | 1,653 | - | 0 | - | 4,058 | - | 0 | - | 0 | N.A. | N.A. | N.A. | 1,124 |
| Shasta | N.A. | N.A. | N.A. | N.A. | N.A. | N.A. | N.A. | N.A. | N.A. | N.A. | N.A. | N.A. | N.A. | N.A. | N.A. | N.A. | N.A. | N.A. | N.A. | N.A. | N.A. | N.A. |

^a^N.A., not applicable.

-, not reported by CSI Phylogeny 1.4.
