# Supplementary material for: Benchmarking Long-Read Assemblers for Genomic Analyses of Bacterial Pathogens Using Oxford Nanopore Sequencing
Source: Int J Mol Sci. 2020 Dec 1;21(23):9161. doi: 10.3390/ijms21239161 (PMC7730629; doi:10.3390/ijms21239161)
Supplement: Supplementary file 1 [file ijms-21-09161-s001.zip › ijms-976706/Supplementary Table S5.docx]

**Supplementary Table S5.** Complete benchmarking universal single-copy orthologs (BUSCOs) of Oxford Nanopore long-read assemblies of bacterial strains with real reads using different long-read assemblers

| Assembler | Complete BUSCOs (%) | | | | | | | | | | |
| --- | --- | --- | --- | --- | --- | --- | --- | --- | --- | --- | --- |
|  | ***P. aeruginosa* CFSAN084950** | ***Bacillus paranthracis* CFSAN068816** | ***E. coli* O157:H7 CFSAN076619** | ***S.* Bareilly CFSAN000189** | ***C. sakazakii* CFSAN068773** | ***C. botulinum* CFSAN034200** | ***L. monocytogenes* CFSAN023468** | ***Staphylococcus aureus* CFSAN007894** | ***Campylobacter coli* CFSAN032805** | ***C. jejuni* NCTC 11168** | **Average** |
| Canu | 42.6 | 40.5 | 18.2 | 34.5 | 28.4 | 6.8 | 31.8 | 33.8 | 1.4 | 2.7 | 24.1 |
| Flye | 31.1 | 1.4 | 20.9 | 10.1 | 10.8 | 0.0 | 2.0 | 0.0 | 0.0 | 0.0 | 7.6 |
| Miniasm/  Racon | 48.0 | 67.6 | 29.1 | 46.6 | 48.0 | 0.7 | 62.2 | 64.9 | 6.1 | 6.8 | 38.0 |
| Raven | 45.3 | 56.8 | 43.9 | 40.5 | 44.6 | 5.4 | 61.5 | 68.9 | 7.4 | 8.8 | 38.3 |
| Redbean | 2.8 | 31.1 | 1.7 | 1.4 | 3.8 | 0.0 | 0.3 | 25.7 | 1.4 | 0.0 | 6.8 |
| Shasta | 29.1 | 27.7 | 27.7 | 3.4 | 24.3 | 4.7 | 34.5 | 32.4 | 0.0 | 2.0 | 18.6 |
| Reference | 100.0 | 98.6 | 100.0 | 99.3 | 100.0 | 98.7 | 100.0 | 100.0 | 84.5 | 88.5 | 97.0 |

^a^N.A., not applicable.
