# Supplementary material for: Benchmarking Long-Read Assemblers for Genomic Analyses of Bacterial Pathogens Using Oxford Nanopore Sequencing
Source: Int J Mol Sci. 2020 Dec 1;21(23):9161. doi: 10.3390/ijms21239161 (PMC7730629; doi:10.3390/ijms21239161)
Supplement: Supplementary file 1 [file ijms-21-09161-s001.zip › ijms-976706/Supplementary Table S6.docx]

**Supplementary Table S6.** Numbers of single nucleotide polymorphisms (SNPs) and indels in Oxford Nanopore long-read assemblies of bacterial strains with real reads using different long-read assemblers, as determined by aligning to their corresponding reference genomes, and expressed as SNPs and indels per one million bp of the reference genomes, respectively

| Assembler | *Pseudomonas aeruginosa* CFSAN084950 | | *Bacillus paranthracis* CFSAN068816 | | *Escherichia coli* O157:H7 CFSAN076619 | | *Salmonella* Bareilly CFSAN000189 | | *Cronobacter sakazakii* CFSAN068773 | | *Clostridium botulinum* CFSAN034200 | | *Listeria monocytogenes* CFSAN023468 | | *Staphylococcus aureus* CFSAN007894 | | *Campylobacter coli* CFSAN032805 | | *Campylobacter jejuni* NCTC 11168 | | Average | |
| --- | --- | --- | --- | --- | --- | --- | --- | --- | --- | --- | --- | --- | --- | --- | --- | --- | --- | --- | --- | --- | --- | --- |
|  | **SNPs** | **Indels** | **SNPs** | **Indels** | **SNPs** | **Indels** | **SNPs** | **Indels** | **SNPs** | **Indels** | **SNPs** | **Indels** | **SNPs** | **Indels** | **SNPs** | **Indels** | **SNPs** | **Indels** | **SNPs** | **Indels** | **SNPs** | **Indels** |
| Canu | 275 | 2,578 | 71 | 3,849 | 665 | 3,609 | 2,108 | 3,652 | 682 | 3,609 | 72 | 7,022 | 5 | 3,619 | 190 | 2,467 | 81 | 11,947 | 50 | 14,180 | 420 | 5,653 |
| Flye | 594 | 3,627 | 77 | 17,410 | 1,018 | 10,064 | 2,517 | 9,986 | 1,206 | 8,331 | 119 | 19,586 | 28 | 18,201 | 164 | 16,871 | 219 | 29,378 | 141 | 29,482 | 608 | 16,294 |
| Miniasm/  Racon | 462 | 1,602 | 190 | 1,804 | 1,010 | 2,075 | 2,814 | 2,116 | 1,031 | 1,962 | 336 | 13,493 | 33 | 1,442 | 219 | 1,086 | 253 | 7,198 | 258 | 12,272 | 661 | 4,505 |
| Raven | 434 | 1,570 | 190 | 1,746 | 980 | 2,059 | 2,799 | 1,971 | 1,026 | 1,867 | 159 | 8,377 | 29 | 1,436 | 207 | 875 | 249 | 7,849 | 222 | 8,302 | 630 | 3,605 |
| Redbean | 537 | 4,164 | 292 | 5,870 | 680 | 6,408 | 2,773 | 8,920 | 722 | 5,740 | 286 | 14,008 | 182 | 6,323 | 505 | 5,236 | 274 | 15,045 | 662 | 6,719 | 691 | 7,843 |
| Shasta | 524 | 5,260 | 172 | 5,085 | 832 | 4,706 | 2,623 | 15,008 | 899 | 4,770 | 72 | 11,478 | 120 | 4,655 | 370 | 4,156 | 236 | 15,667 | 685 | 17,170 | 653 | 8,796 |
