# Supplementary material for: Benchmarking Long-Read Assemblers for Genomic Analyses of Bacterial Pathogens Using Oxford Nanopore Sequencing
Source: Int J Mol Sci. 2020 Dec 1;21(23):9161. doi: 10.3390/ijms21239161 (PMC7730629; doi:10.3390/ijms21239161)
Supplement: Supplementary file 1 [file ijms-21-09161-s001.zip › ijms-976706/Supplementary Table S8.docx]

**Supplementary Table S8.** Plasmids of bacterial strains with low-quality reads compared to their corresponding reference genomes, as predicted based on their Oxford Nanopore long-read assemblies using different long-read assemblers^a^

| Assembler | Plasmid | | | | |
| --- | --- | --- | --- | --- | --- |
|  | ***Escherichia coli* O157:H7 Sakai** | ***Bacillus anthracis* Ames Ancestor** | ***Salmonella* Typhimurium LT2** | ***Cronobacter sakazakii* ATCC 29544** | ***Staphylococcus aureus* TW20** |
| Canu | -^b^ | - | - | - | - |
| Flye | - | - | - | - | - |
| Miniasm/Racon | - | - | - | - | - |
| Raven | - | - | - | - | rep7 |
| Redbean | - | - | - | - | - |
| Shasta | - | - | - | - | - |
| Reference | IncFIB (AP001918) | rep3 | IncFIB(S)  IncFII(S) | IncFIB (pCTU3) | rep7  rep20  rep21 |

^a^Plasmids were not identified in any Oxford Nanopore long-read assemblies of *Pseudomonas aeruginosa* PAO1, *Klebsiella variicola* DSM 15968, *Clostridium botulinum* CDC_1632, *Listeria monocytogenes* EGD-e, and *Campylobacter jejuni* NCTC 11168.

^b^-, not detected.
