# Supplementary material for: Benchmarking Long-Read Assemblers for Genomic Analyses of Bacterial Pathogens Using Oxford Nanopore Sequencing
Source: Int J Mol Sci. 2020 Dec 1;21(23):9161. doi: 10.3390/ijms21239161 (PMC7730629; doi:10.3390/ijms21239161)
Supplement: Supplementary file 1 [file ijms-21-09161-s001.zip › ijms-976706/Supplementary Table S9.docx]

**Supplementary Table S9.** Plasmids of bacterial strains with real reads compared to their corresponding reference genomes, as predicted based on their Oxford Nanopore long-read assemblies using different long-read assemblers^a^

| Assembler | Plasmid | | |
| --- | --- | --- | --- |
|  | ***Escherichia coli* O157:H7 CFSAN076619** | ***Salmonella* Bareilly CFSAN000189** | ***Staphylococcus aureus* CFSAN007894** |
| Canu | - ^b^ | IncFII (S) | rep7  rep20 |
| Flye | - | IncFII (S) | rep20 |
| Miniasm/Racon | - | IncFII (S) | rep7  rep20 |
| Raven | - | IncFII (S) | rep7  rep20 |
| Redbean | - | IncFII (S) | rep7 |
| Shasta | - | - | - |
| Reference | IncFIB(AP001918) | IncFII (S) | rep20 |

^a^Plasmids were not identified in any Oxford Nanopore long-read assemblies of *Pseudomonas aeruginosa* CFSAN084950, *Bacillus paranthracis* CFSAN068816, *Cronobacter sakazakii* CFSAN068773, *Clostridium botulinum* CFSAN034200, *Listeria monocytogenes* CFSAN023468, *Campylobacter coli* CFSAN032805, and *Campylobacter jejuni* NCTC 11168.

^b^-, not detected.
